# Genome‐scale CRISPR–Cas9 screen identifies *PAICS* as a therapeutic target for *EGFR* wild‐type non‐small cell lung cancer

**DOI:** 10.1002/mco2.483

**Published:** 2024-03-09

**Authors:** Yufeng Li, Lingyun Zhu, Jiaqi Mao, Hongrui Zheng, Ziyi Hu, Suisui Yang, Tianyu Mao, Tingting Zhou, Pingping Cao, Hongshuai Wu, Xuerong Wang, Jing Wang, Fan Lin, Hua Shen

**Affiliations:** ^1^ Department of Medical Oncology The First Affiliated Hospital of Nanjing Medical University Nanjing Jiangsu China; ^2^ Department of Medical Oncology The Affiliated Sir Run Run Hospital of Nanjing Medical University Nanjing Jiangsu China; ^3^ Department of Cell Biology School of Basic Medical Sciences, Nanjing Medical University Nanjing Jiangsu China; ^4^ Department of Orthopedics Taizhou Hospital of Zhejiang Province Affiliated to Wenzhou Medical University Zhejiang China; ^5^ Department of Pharmacology Nanjing Medical University Nanjing Jiangsu China; ^6^ Institute for Brain Tumors & Key Laboratory of Rare Metabolic Diseases, Nanjing Medical University Nanjing Jiangsu China; ^7^ Department of Gastroenterology The First Affiliated Hospital and College of Clinical Medicine of Henan University of Science and Technology Luoyang Henan China

**Keywords:** cell cycle, DNA damage, *EGFR* wild‐type NSCLC, *PAICS*

## Abstract

Epidermal growth factor receptor‐targeted (*EGFR*‐targeted) therapies show promise for non‐small cell lung cancer (NSCLC), but they are ineffective in a third of patients who lack *EGFR* mutations. This underlines the need for personalized treatments for patients with *EGFR* wild‐type NSCLC. A genome‐wide CRISPR/Cas9 screen has identified the enzyme phosphoribosylaminoimidazole carboxylase/phosphoribosylaminoimidazole succinocarboxamide synthetase (*PAICS*), which is vital in de novo purine biosynthesis and tumor development, as a potential drug target for *EGFR* wild‐type NSCLC. We have further confirmed that PAICS expression is significantly increased in NSCLC tissues and correlates with poor patient prognosis. Knockdown of *PAICS* resulted in a marked reduction in both in vitro and in vivo proliferation of *EGFR* wild‐type NSCLC cells. Additionally, *PAICS* silencing led to cell‐cycle arrest in these cells, with genes involved in the cell cycle pathway being differentially expressed. Consistently, an increase in cell proliferation ability and colony number was observed in cells with upregulated *PAICS* in *EGFR* wild‐type NSCLC. *PAICS* silencing also caused DNA damage and cell‐cycle arrest by interacting with DNA repair genes. Moreover, decreased IMPDH2 activity and activated PI3K–AKT signaling were observed in NSCLC cells with *EGFR* mutations, which may compromise the effectiveness of *PAICS* knockdown. Therefore, *PAICS* plays an oncogenic role in *EGFR* wild‐type NSCLC and represents a potential therapeutic target for this disease.

## Graphical abstract

Genome‐wide CRISPR–Cas9 screening identified *PAICS* as a potential target for *EGFR* wild‐type NSCLC. Increased *PAICS* expression in NSCLC tissues correlates with poor prognosis. *PAICS* knockdown markedly reduces in vitro and in vivo proliferation of *EGFR* wild‐type NSCLC cells, inducing cell‐cycle arrest through interaction with DNA repair genes. This highlights *PAICS* as an oncogenic factor and a potential therapeutic target for *EGFR* wild‐type NSCLC.

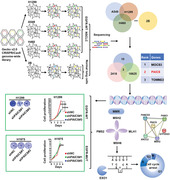

## 1 INTRODUCTION

Lung cancer is a leading cause of cancer‐related mortality worldwide, and the prognosis for patients is often poor due to late‐stage diagnosis.^1^ Over recent decades, treatment strategies for advanced NSCLC, which comprises 85% of all lung cancer cases, have undergone significant changes.^2^ Targeted therapy based on small molecules has emerged as a promising approach and has substantially improved treatment outcomes for NSCLC patients carrying targetable molecular alterations, including *EGFR* activating mutations, anaplastic lymphoma kinase (*ALK*) translocations, c‐ros oncogene 1 (*ROS1*) translocation,^3^, ^4^, ^5^ and so on. Targeted therapy has already been recommended as the first‐line treatment for advanced NSCLC by the American Society of Clinical Oncology, the European Society of Oncology, and National Comprehensive Cancer Network, due to its superior efficacy and lower toxicity when compared with traditional chemotherapeutic agents.^6^, ^7^, ^8^, ^9^

Osimertinib, one of the most successful EGFR tyrosine kinase inhibitors (EGFR‐TKIs), has demonstrated significant improvements in median progression‐free survival (PFS) and overall survival (OS) for NSCLC patients with *EGFR* mutations.^10^, ^11^ In 2015, the United States Food and Drug Administration granted approval for the treatment of patients with *EGFR* T790M‐mutated NSCLC. However, a considerable proportion of NSCLC patients, particularly 50% of Asian and 80% of Western patients, do not benefit from *EGFR*‐targeted treatments unless they present with *EGFR*‐sensitizing mutations.^12^ As a result, chemotherapy remains the standard of care for *EGFR* wild‐type NSCLC patients in routine clinical practice. Hence, the development of novel personalized molecular‐targeted therapies is urgently required to enhance survival and improve the quality of life for these patients.

CRISPR/Cas9‐based functional genomic screening is widely utilized for systematic exploration of gene function and characterization of their role in cellular fitness on a genome‐wide scale.^13^ This approach offers high specificity and generates stable phenotypes by creating null alleles, rather than partial gene knockdown. Importantly, CRISPR/Cas9 screens have demonstrated success in identifying novel drug targets for various cancers.^14^, ^15^, ^16^ In this study, we present genome‐scale CRISPR/Cas9 fitness screens in several *EGFR* wild‐type NSCLC cells to find genes that are essential for cell survival. As a result, *PAICS*, a putative bifunctional enzyme involved in histidine de novo purine biosynthesis with both 5‐aminoimidazole ribonucleotide carboxylase and 4‐(N‐succinylcarboxamide)−5‐aminoimidazole ribonucleotide synthetase (SAICARs) activities, was identified as a new therapeutic target for *EGFR* wild‐type NSCLC.^17^ *PAICS* has been shown to exhibit high expression levels across various cancer types and is a prognostic indicator for unfavorable patient outcomes.^18^, ^19^, ^20^ Nevertheless, the precise biological functions and underlying mechanisms of *PAICS* in *EGFR* wild‐type NSCLC are not well established.

In the current investigation, we meticulously explored the contributory role of PAICS in the biology of *EGFR* wild‐type NSCLC and delved into the underlying molecular mechanisms. Our analyses unequivocally demonstrated an elevated expression profile of PAICS in NSCLC histological specimens, with its augmented expression being inextricably associated with a less favorable prognostic trajectory in NSCLC patients. Notably, targeted attenuation of PAICS robustly curtailed the proliferative propensity and growth dynamics of *EGFR* wild‐type NSCLC cellular models, both in vitro assays and in vivo murine xenograft frameworks. Of paramount significance, the silencing of *PAICS* orchestrated a pronounced induction of cell‐cycle quiescence and incurred DNA integrity perturbations, ostensibly through its interactions with a cadre of DNA repair‐centric gene entities. Comprehensive elucidation of the specific roles of *PAICS* in *EGFR* wild‐type NSCLC biology would facilitate further investigations aimed at identifying potential therapeutic targets.

## 2 RESULTS

### 2.1 Genome‐wide CRISPR/Cas9 screens identified *PAICS* as a new drug target for *EGFR* wild‐type NSCLC

**FIGURE 1:**
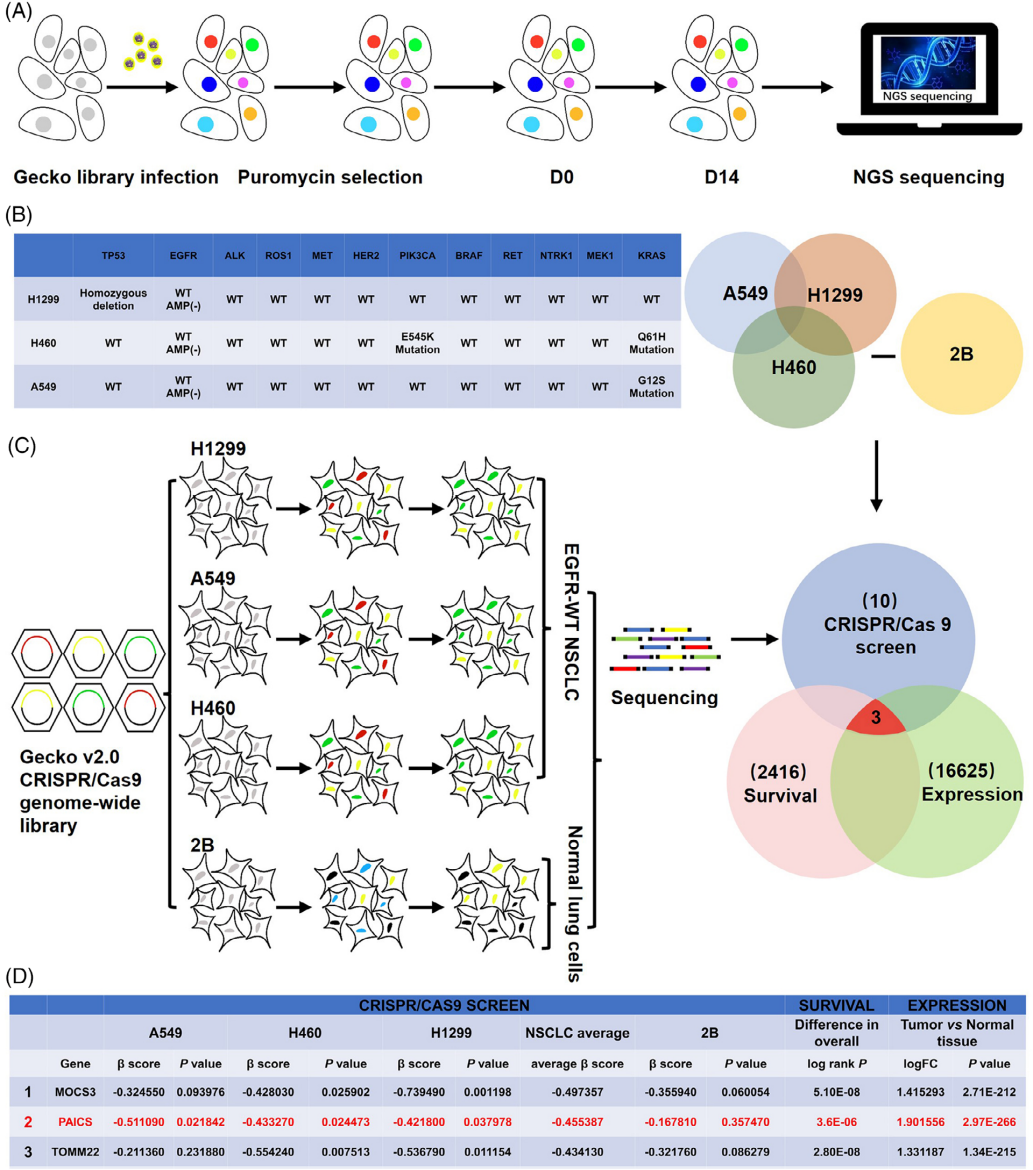
Genome‐wide CRISPR/Cas9 screens combined with the TCGA database identified *PAICS* as a promising therapeutic target for *EGFR*‐WT NSCLC. (A) Schematic diagram of the CRISPR/Cas9 screens. (B) The mutated status of lung cancer driver oncogenes in H1299, A549, and H460 cell lines. (C) The flowchart shows the screening process. (D)Three genes were screened out. Survival data come from Kaplan‐Meier: MOCS3(206141_‐_at); PAICS (214664_‐_at); TOMM22(217960_‐_s_‐_at).

Genome‐wide CRISPR/Cas9 screen was performed in H460, H1299, and A549 cell lines which harbor wild‐type *EGFR* genes, and BEAS‐2B cells as control (Figure 1A). The status of some common driver gene mutations of H460, H1299, and A549 is listed in Figure 1B. Three genes were screened out according to the following procedures (Figure 1C): (1) at least one *β* score less than 0 among A549/H460/H1299 cells screened by CRISPR/CAS9; (2) at least one *p* value less than 0.05 among A549/H460/H1299 cells screened by CRISPR/CAS9; (3) *p* value more than 0.05 in BEAS‐2B cells screened by CRISPR/CAS9; (4) *p* less than 0.05 in Kaplan–Meier analysis of OS of NSCLC patients with the gene expression; (5) logFC more than 1 in NSCLC tumor versus normal tissues. The three genes were ranked based on NSCLC average *β* score in CRISPR/CAS9 screen (Figure 1D).

### 2.2 *PAICS* genetic alteration analysis data

We explored the PAICS genetic alterations in human tumor samples. The frequency of PAICS genetic alteration (∼4%) is high in Glioblastoma multiforme, Diffuse glioma and Lung squamous cell carcinoma, with “amplification” as the primary type of alteration. Melanoma and Uterine endometrioid carcinoma had the highest incidence of “mutation,” with the frequency of ∼3% and ∼2%, respectively (Figure S1A). We have presented additional mutations and their locations within PAICS (Figure S1B). We did not identify a predominant type of genetic alteration, and their locations appeared to be somewhat sporadic. Among these mutations, the highest frequency was observed for the R222*/Q mutation, which occurred in five out of 189138 patients (Figure S1B). Furthermore, we identified that in cancer patients with genetic alteration of PAICS showed a poor prognosis in OS (*p* = 5.08e−12), PFS (*p* = 5.16e−3), disease‐specific survival (DSS) (*p* = 3.003e−3), and disease‐free survival (DFS) (*p* = 0.0131), compared with patients without PAICS alterations (Figure S1C).

We used the TIMER2 to study the differential expression of PAICS between tumor and adjacent normal tissues for tumors represented in the TCGA repository (Figure S1D). The expression level of PAICS in the tumor tissues of BLCA, BRCA, COAD, ESCA, GBM, HNSC, LIHC, LUAD, LUSC, PRAD, READ, STAD, THCA, UCEC (*p* < 0.001), CESC, SKCM (*p* < 0.01), CHOL, and PAAD (*p* < 0.05) is higher than the corresponding control tissues. Remarkably, only a few tumor types showed no differential expression (e.g. KICH, KIRC, and PCPG). In contrast, PAICS showed lower expression only in KIRP (*p* < 0.001) relative to the corresponding control tissues (Figure S1D).

### 2.3 *PAICS* is upregulated in NSCLC tissues and correlated with the prognosis of patients with NSCLC

**FIGURE 2:**
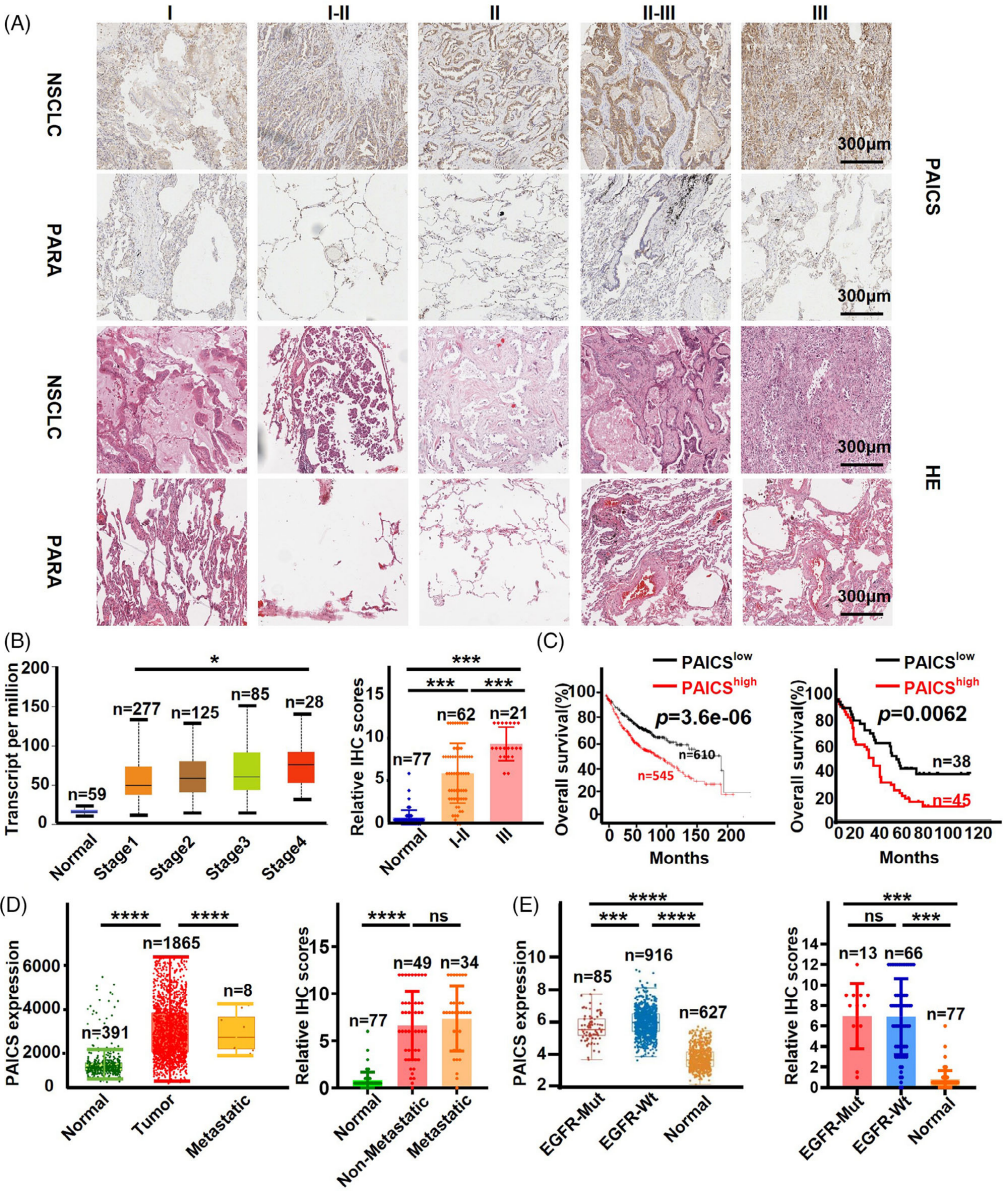
*PAICS* upregulated and served as a prognostic factor in NSCLC. (A) IHC staining of *PAICS* and HE staining on the tissue microarray from 83 lung adenocarcinoma tissues. (B) *PAICS* expression in NSCLC tissues in TCGA and GTEx data (left) and microarray (right). (C) Kaplan–Meier analysis shows the OS of NSCLC patients based on *PAICS* expression. (D) Relationship between *PAICS* expression and NSCLC metastasis (left: TCGA; right: microarray). (E) Graphical representation of the relationship between the *PAICS* expression and NSCLC patient's *EGFR* status (left: TCGA; right: microarray). Data are represented as means ± SD. ****p < 0.0001, ***p < 0.001, and *p < 0.05.

Patient‐derived tissue microarray analysis on *PAICS* in 83 lung adenocarcinoma samples and their corresponding adjacent normal tissues was performed. *PAICS* protein significantly elevated in tumor specimens revealed by IHC staining (Figures 2A and B, right). Consistent results were also observed in the analysis results of public database (Figure 2B, left). The upregulated *PAICS* expression was significantly associated with a poorer prognosis and a shorter OS in both public database and the microarray data (Figure 2C). At the same time, *PAICS* level were related to tumor metastasis, *EGFR* status and gender in NSCLC patients based on TCGA database (Figures 2D, E, and S2A, left), whereas uncorrelated with tumor metastasis, *EGFR* status and gender from microarray analysis (Figures 2D, E, and S2A, right). It is likely due to the small sample size of *EGFR*‐positive subsamples in the microarray, which causes inaccurate statistical results. In addition, using the UALCAN web tool, *PAICS* expression were associated with TP53 mutation status and smoking habits (Figure S2B), whereas not correlated with age (Figure S2C). The above findings suggest that high level of *PAICS* facilitates NSCLC carcinogenesis, and the level of PAICS might be a valuable prognostic factor in NSCLC patients.

### 2.4 Knockdown of *PAICS* attenuates the malignant characteristics of tumor in vitro

**FIGURE 3:**
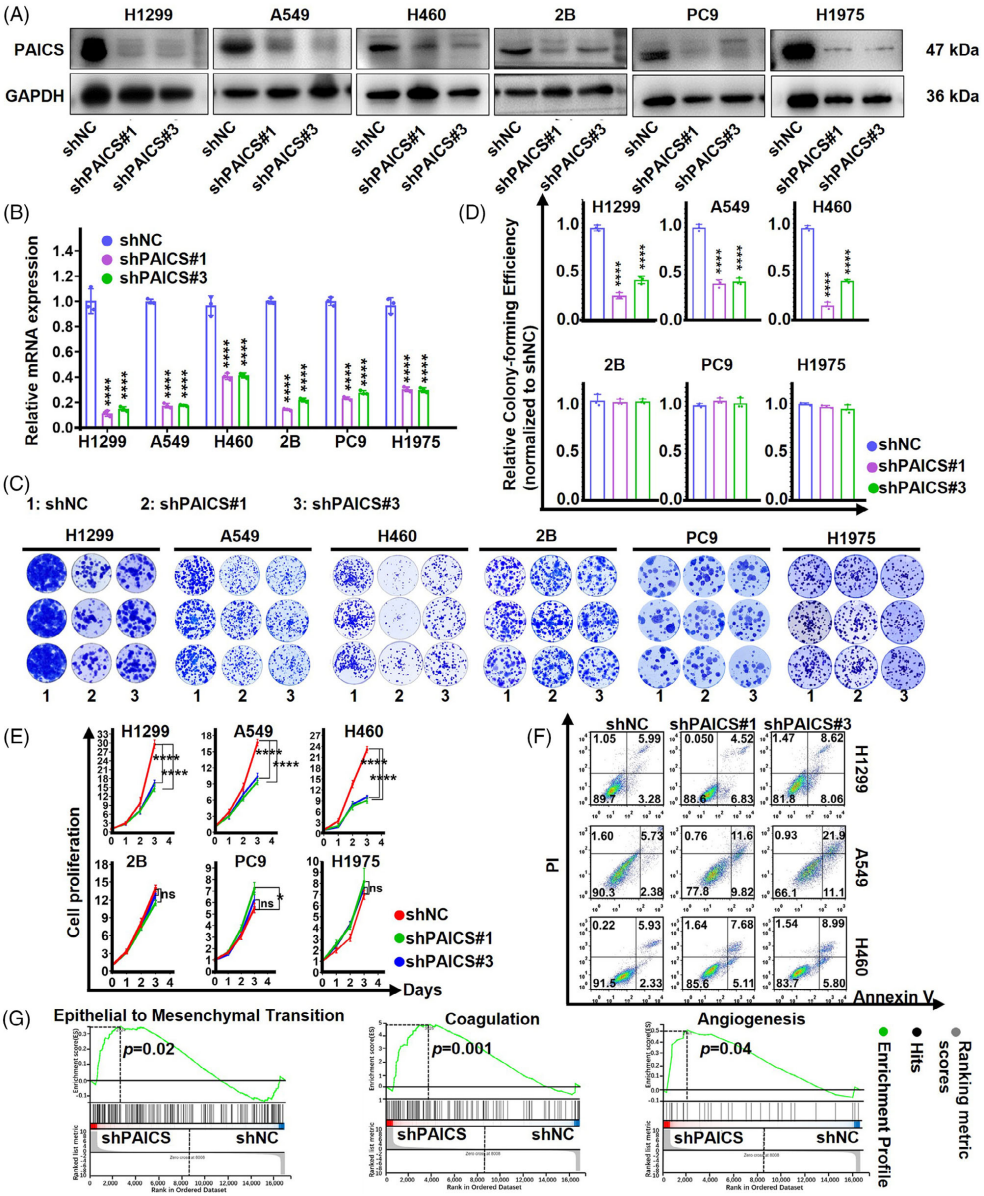
*PAICS* deficiency inhibited *EGFR*‐WT NSCLC cell proliferation in vitro. (A) *PAICS*‐knockdown efficiency detected by Western blot in lung cancer cells. (B) *PAICS*‐knockdown efficiency detected by qRT‐PCR in lung cancer cells. (CD) Colony formation (C) and Clone quantification (D) of above‐mentioned cells (*n* = 3). (E) Cell viability of shNC and shPAICS cells confirmed by CCK8 assays. (F) Apoptosis of shNC and shPAICS cells detected by Flow cytometric analysis. (G) GSEA analysis for EMT, coagulation and angiogenesis. Data are represented as means ± SD (*n* = 3). ****p < 0.0001, ***p < 0.001, **p < 0.01, *p < 0.05.

To validate the screening results, the role of *PAICS* in NSCLC cell proliferation was investigated. *PAICS* in the *EGFR* wild‐type (A549, H460, and H1299), *EGFR* mutant (PC9 and H1975) NSCLC cell lines and BEAS‐2B cells were knockdown using short hairpin RNA‐encoding lentiviruses (shNC, shPAICS#1, shPAICS#3). The status of some common driver gene mutations of PC9 and H1975 is listed in Figure S3A.Then, PAICS protein and mRNA level was successfully reduced after the shRNA transfection (Figures 3A and B). A marked reduction of the colony number was identified in *PAICS* knockdown A549, H460 and H1299 cells, whereas unremarkable colony number change was observed in BEAS‐2B, PC9, and H1975 cells following *PAICS* knockdown (Figures 3C and D). Consistently, cell viability of A549, H460, and H1299 cells significantly decreased after *PAICS* knockdown, whereas cell viability of BEAS‐2B, PC9, and H1975 showed no obvious change (Figure 3E). In addition, an increased percentage of apoptosis in *PAICS*‐knockdown A549, H460, and H1299 cells was confirmed by flow cytometry (Figure 3F). Furthermore, we engineered an elevation of PAICS expression in BEAS‐2B, H1299, A549, and H460 cellular models through meticulous transfection of PAICS‐OE lentivirus. After the implementation of *PAICS*‐OE transfection, there was a discernible augmentation in both PAICS protein and mRNA levels (Figures S3B and C). The cell viability of BEAS‐2B, H1299, A549, and H460 cells exhibited a significant increment post‐PAICS overexpression (Figure S3D). Consistently, a pronounced enhancement in colony number was manifested in PAICS‐upregulated BEAS‐2B, H1299, A549, and H460 cellular cohorts (Figures S3E and F). Moreover, based on GSEA analysis from the RNA‐sequencing database, epithelial to mesenchymal transition (EMT), coagulation, and angiogenesis, the three major characteristics related to malignant tumors, decreased in PAICS deficiency *EGFR* wild‐type NSCLC cells (Figure 3G). To sum up, knocking down *PAICS* only inhibited *EGFR* wild‐type NSCLC carcinogenesis in vitro.

Next, we identified that the level of Inosine‐5′‐monophosphate dehydrogenase (IMPDH2), a key enzyme in the de novo purine biosynthesis pathway, is significantly lower in *EGFR* mutant NSCLC cells (PC9 and H1975) than *EGFR* wild‐type NSCLC cells (H1299 and A549) (*p* < 0.0001;, Figure S4A). In addition, the p‐AKT protein level obviously higher in *EGFR* mutant NSCLC cells compared with *EGFR* wild‐type NSCLC cells after *PAICS* gene was knockdown (Figure S4B). Furthermore, treating *EGFR* mutant NSCLC cells with a pan‐PI3K inhibitor BKM120 led to a significant drop of the proliferation ability of *PAICS* knockdown cells (Figure S4C), suggesting the activated PI3K‐AKT signaling could compromise the effect of PAICS knockdown.

### 2.5 *PAICS* knockdown induces cell‐cycle arrest in *EGFR* wild‐type NSCLC cells

**FIGURE 4:**
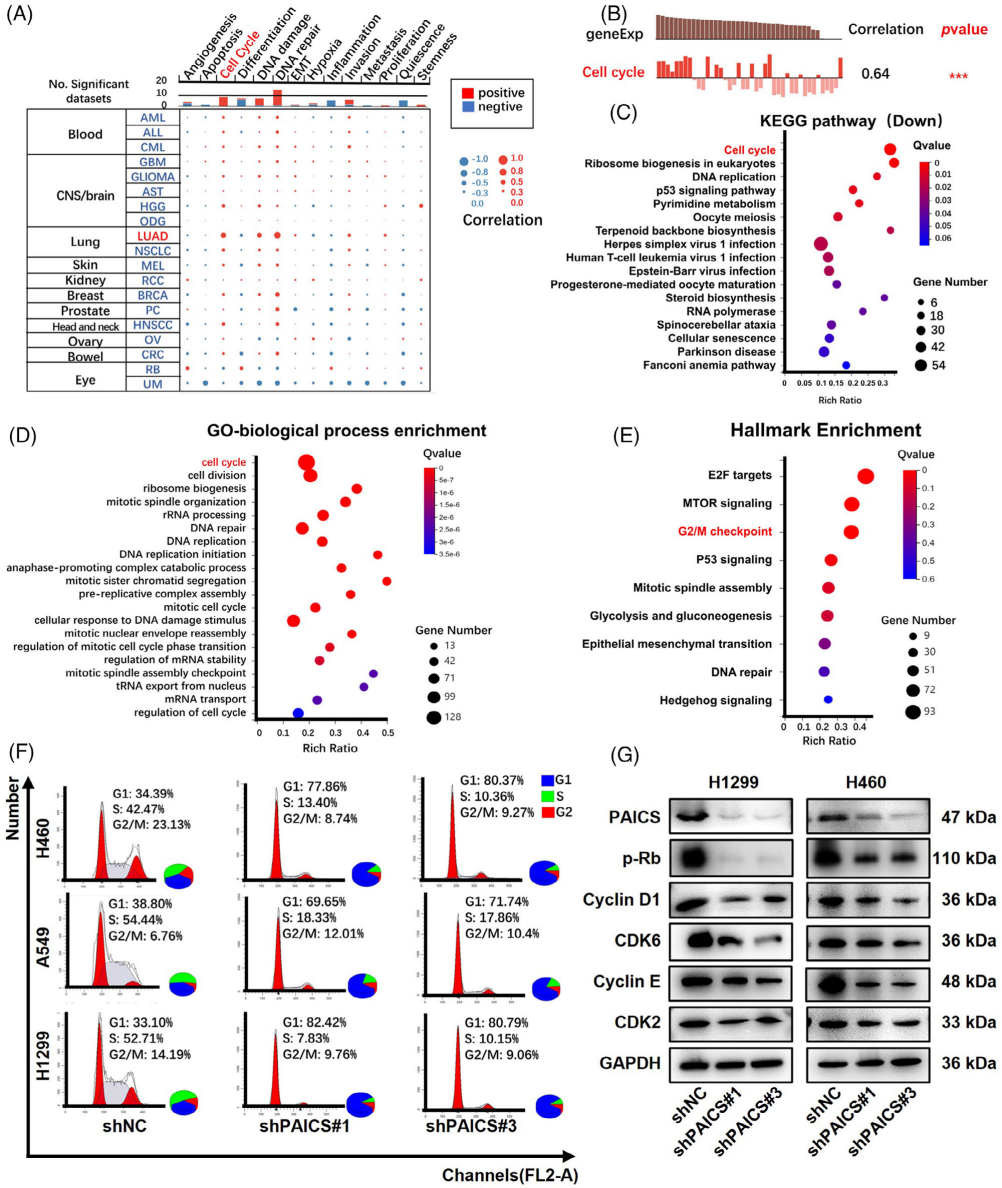
*PAICS* silencing obviously induced cell‐cycle arrest in *EGFR*‐WT NSCLC cells. (A) *PAICS* was primarily involved in the regulation of the cell cycle in NSCLC indicated by single‐cell analysis from CancerSEA. (B) *PAICS* mRNA level was positively correlated with cell cycle demonstrated by Data from Guillaumet‐Adkins A. Genome Biol. 2017 (PDX_LUAD) (*n* =   42). (C and D) KEGG pathway and GO‐biological process analysis indicated that the cell cycle, DNA repair, and DNA replication pathway were down‐regulated after knocking down *PAICS*. (E) MSigDB H (hallmark) analysis identified nine signatures (*Q* value ≤ 0.05) that were differentially expressed once silencing *PAICS*. (F) Cell cycle distribution of shNC‐ and shPAICS‐NSCLC cells. (G) CDK2, CDK6, Cyclin D1, Cyclin E, and p‐Rb protein levels in shNC‐ and shPAICS‐NSCLC cells.

To explore the functions of *PAICS*, CancerSEA was first used for single‐cell analysis. The results indicated that *PAICS* was primarily involved in regulating the cell cycle (Figures 4A, B, and S5A). Consistently, the KEGG pathway and GO‐biological process analysis from the RNA‐sequencing database indicated that the downregulated genes were enriched in the regulation of the cell cycle (Figures 4C and D). Based on MSigDB (hallmark) analysis, among the nine significantly expressed hallmark gene sets, four cell cycle gene sets were enriched, G2/M checkpoint, E2F targets, mTOR signaling, and mitotic, consistent with dysregulated cell proliferation (Figure 4E). Furthermore, *PAICS* silencing significantly increased the proportion of cells at G‐phase and reduced the proportion of cells at S‐phase, suggesting that *PAICS* knockdown indeed blocked the cell cycle (Figure 4F). Consistently, *PAICS* knockdown decreased the level of the cell cycle‐related genes, including CDK2, CDK6, Cyclin D1, Cyclin E, and p‐Rb expression in PAICS‐knockdown *EGFR* wild‐type NSCLC cell lines (Figure 4G). Therefore, *PAICS* knockdown‐induced cell cycle arrest might play an important role in *EGFR* wild‐type NSCLC cells.

### 2.6 *PAICS* knockdown blocks the cell cycle by inducing DNA damage

**FIGURE 5:**
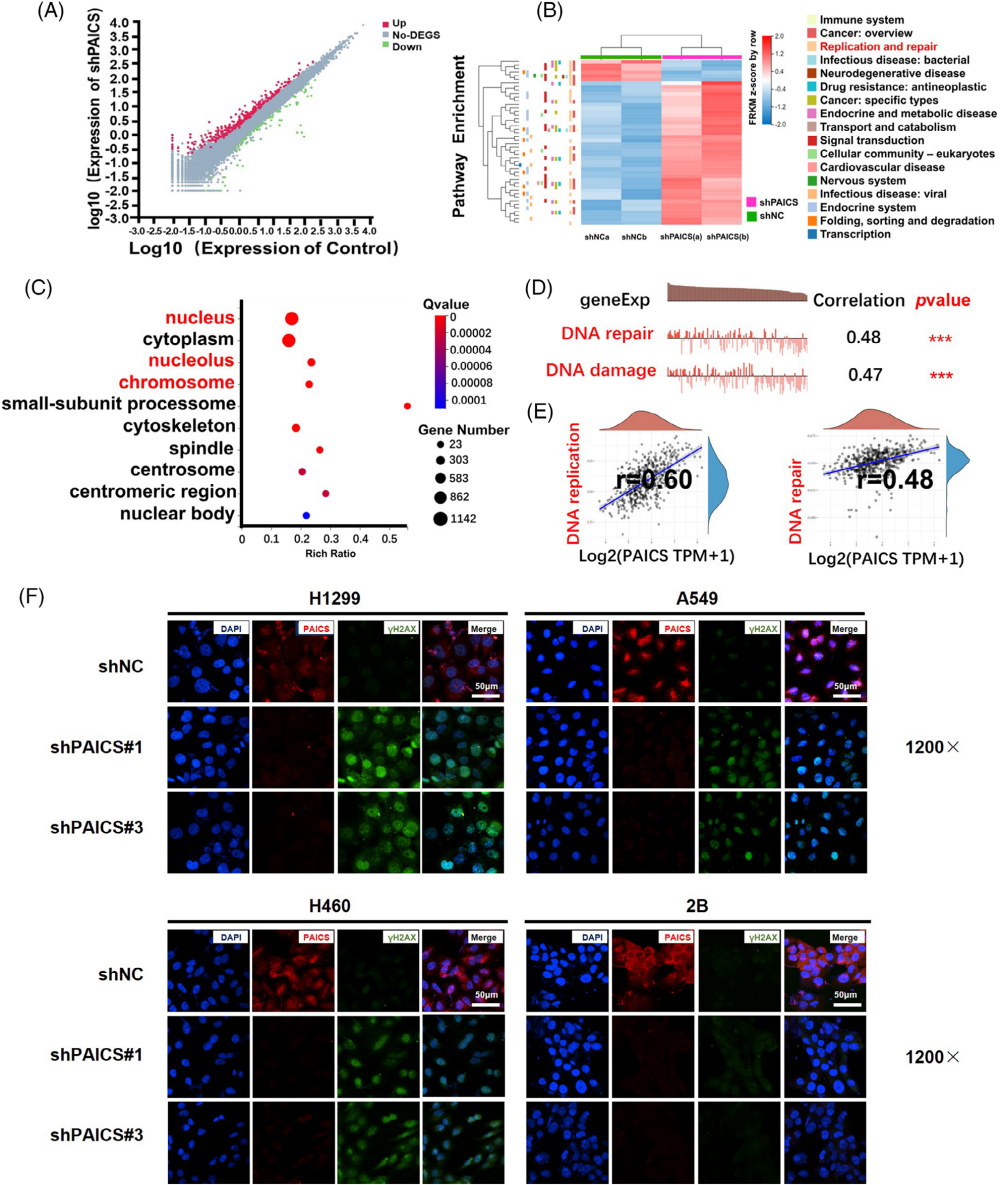
*PAICS* knockdown blocks the cell cycle by inducing DNA damage. (A–C) Based on RNA‐sequencing (RNA‐seq) analysis, (A) 2420 differentially expressed genes (DEGs) with 1216 upregulated and 1204 downregulated after *PAICS* knockdown. (B) 2420 DEGs were shown as heatmaps. (C) GO‐cellular component analysis of DEGs. (D) *PAICS* mRNA expression was positively correlated with DNA repair and damage, confirmed by Data from Kim KT. Genome Biol. 2015 (PDX) (No. of cells = 126). (E) *PAICS* mRNA expression was related to the DNA repair (*p* = 8.54e−31, *r* = 0.48) and replication (*p *= 9.04e−51, *r* = 0.6) pathway confirmed by Data from the TCGA database (*n *= 516). (F) Immunofluorescence analysis of the formation of *γH2AX* foci in shNC‐ and shPAICS‐NSCLC and BEAS‐2B cell lines. Scale bar 50 µm.

To investigate the mechanisms of *PAICS* in promoting NSCLC growth, we performed RNA‐seq analysis of H1299‐shPAICS cells. *PAICS* knockdown resulted in differential expression of various genes, including 1216 upregulated genes and 1204 downregulated genes (|log2FC|≥0, *Q* value ≤ 0.05) (Figures 5A and B). By conducting Gene set analysis with the KEGG Pathway Term Level2 method, differentially expressed genes were enriched in DNA repair and replication pathways (Figure 5B). Consistently, differentially expressed genes were in the nucleus confirmed by GO‐cellular component analysis (Figure 5C). Based on the single‐cell analysis, *PAICS* level was significantly related to DNA damage, replication, and repair (Figures 5D and E). What is more, knockdown of *PAICS* led a marked increase in γH2AX foci formation in *EGFR* wild‐type NSCLC cells (H1299, A549, and H460 cells) rather than BEAS‐2B cells, which confirmed that *PAICS* knockdown induced DNA damage (Figures 5F and S5B). In conclusion, *PAICS* led to cell cycle arrest in *EGFR* wild‐type NSCLC cells by inducing DNA damage response.

### 2.7 Knockdown of *PAICS* inhibits NSCLC carcinogenesis in vivo

**FIGURE 6:**
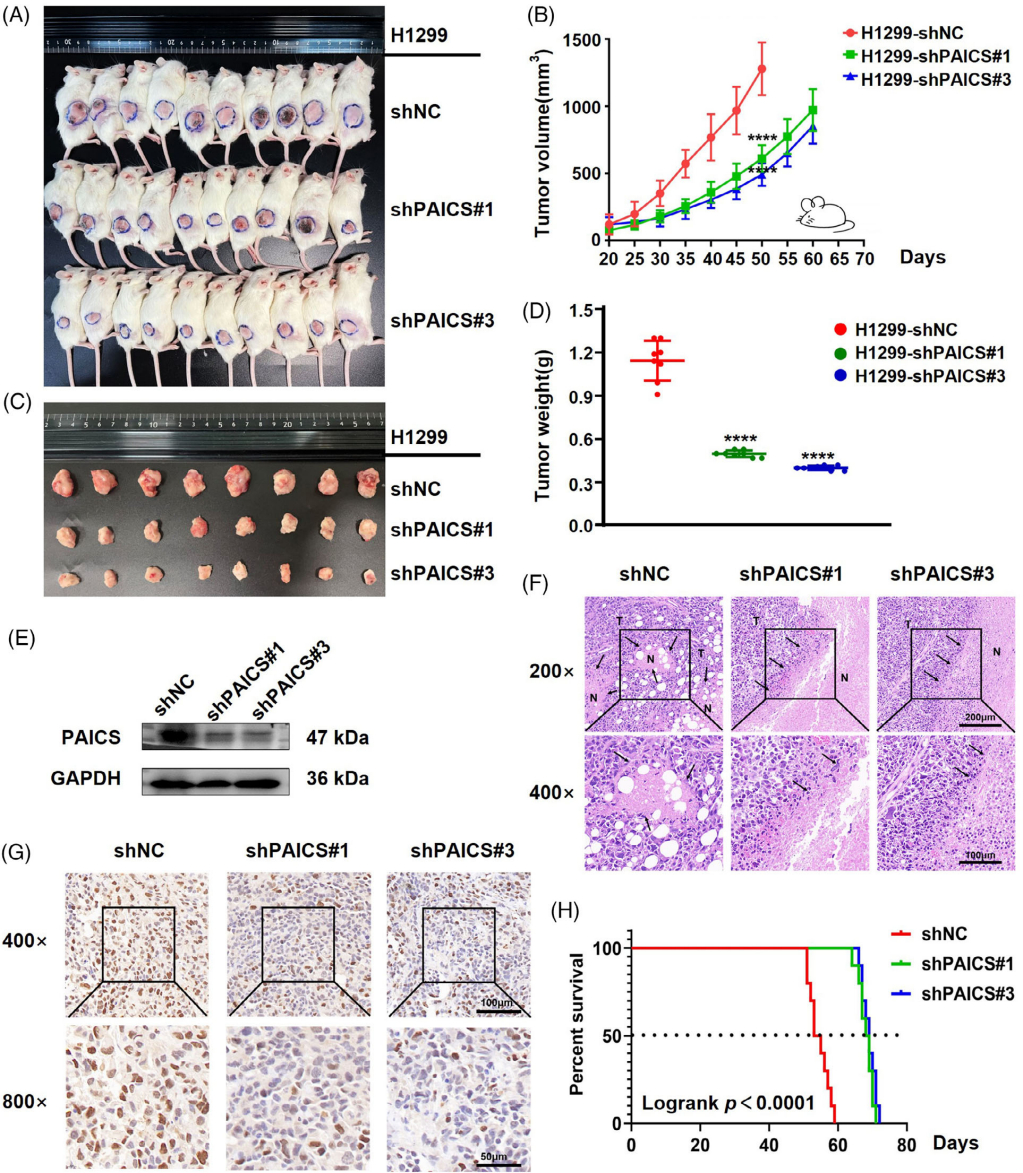
*PAICS* promotes NSCLC carcinogenesis in vivo. (A) The image shows xenotransplanted tumor mice. (B) Tumor volume was measured at the indicated days after mice were transplanted. (C) Xenograft tumors were removed. (D) Measure of the weight of tumor after they were removed. (E) Expression of PAICS in Subcutaneous tumors was exhibited by Western blot. (F and G) HE and Ki67 IHC staining of xenograft tumors were shown. (H) The survival time of xenografted tumor mice was monitored.

The effect of *PAICS* on the tumor in vivo was further evaluated by establishing a subcutaneous xenograft tumor model in immunodeficient mice using PAICS‐knockdown and the control H1299 cells. There was no difference in the body weight between PAICS knockdown mice and the control group (Figure S5C). As expected, tumor growth was dramatically slower in two *PAICS*‐knockdown groups (shPAICS#1, shPAICS#3) compared with the control group (shNC) (Figures 6A and B and Tables 1 and 2). The volumes of tumor in *PAICS*‐knockdown groups were significantly smaller, and the weights were significantly lower than those in the control group (Figures 6C and D and Tables 1 and 2). PAICS expression in subcutaneous tumor exhibited a significant decrease in PAICS knockdown group (Figure 6E). HE staining of the fixed tumor tissue showed that the nuclei were large and deeply stained, the atypia was obvious, and the pathological mitosis was common, which indicated the tumor tissue was obtained. Less aggressive and invasive growth of cells were observed in *PAICS* knockdown tumors compared with shNC. The front edge of the *PAICS* knockdown tumor was clearly visible. In contrast, the growth of the control tumor appeared to be more aggressive since a clear tumor edge was hard to be found (Figure 6F). In addition, a significant decrease was identified in the positive rate of Ki67 in the PAICS‐knockdown group by IHC analysis (Figures 6G and S5D). Moreover, the survival time of mice injected with H1299‐shPAICS was significantly better than that of mice injected with shNC demonstrated by the survival curve (Figure 6H). Therefore, knockdown of *PAICS* significantly inhibited tumor growth in vivo.

**TABLE 1:** Subcutaneous tumor volume of mice.

Time (days)	Tumor volume (mm^3^)									
**H1299‐shNC**										
0	1#	2#	3#	4#	5#	6#	7#	8#	9#	10#
20	238.05	210.94	55.69	98.31	62.50	90.75	46.23	62.50	222.91	108.00
25	333.90	329.25	88.48	153.60	171.50	140.40	98.31	137.31	261.56	256.00
30	503.61	457.65	326.40	357.64	296.35	284.00	225.26	228.27	425.25	402.18
35	605.05	732.05	622.91	613.84	589.84	488.45	400.95	428.69	655.99	578.81
40	804.65	1031.25	739.26	906.304	744.15	622.91	525.00	530.60	972.00	800.81
45	1000.80	1155.07	1106.63	1021.21	881.37	752.64	740.60	734.22	1183.00	1098.50
50	1461.25	1400.77	1333.08	1421.00	1093.75	932.91	1132.63	1098.50	1482.05	1431.64
55	–	–	–	–	1280.66	1225.25	1441.73	1482.55	–	–

H1299‐shPAICS#1										
0	11#	12#	13#	14#	15#	16#	17#	18#	19#	20#
20	102.85	55.18	94.22	89.24	90.75	39.75	121.00	75	50.63	32.46
25	129.60	88.48	134.95	117.00	124.65	62.50	188.42	132.10	111.60	74.43
30	205.80	175.07	193.55	191.56	183.75	111.60	274.44	163.84	166.46	130.24
35	311.65	273.78	266.81	272.00	262.40	194.51	355.20	215.60	225.00	191.56
40	433.35	406.56	382.93	402.04	340.61	256.00	496.86	275.70	295.86	300.80
45	575.00	550.00	543.744	520.00	465.75	368.55	614.66	370.88	388.80	387.20
50	704.11	739.26	729.00	611.89	595.51	485.15	681.46	478.33	546.21	531.61
55	876.10	907.20	970.03	740.60	715.01	612.52	912.52	635.42	665.50	704.11
60	1053.26	1089.54	1264.76	878.40	864.00	807.65	1143.07	807.65	893.10	914.40
65	1359.46	1461.89	–	1106.95	1016.06	1124.05	1440.60	1016.06	1176.12	1139.91
70	–	–	–	1441.73	–	–	–	–	1524.31	–

H1299‐shPAICS#3										
0	21#	22#	23#	24#	25#	26#	27#	28#	29#	30#
20	111.93	111.01	122.79	219.38	186.62	154.87	45.86	85.18	53.14	87.64
25	127.83	122.40	139.54	256.00	199.84	171.50	117.00	94.77	61.37	93.75
30	153.76	138.92	156.78	285.89	213.75	219.49	161.84	116.06	87.12	109.55
35	215.60	275.68	252.05	344.61	318.03	269.00	231.23	158.44	116.25	153.00
40	292.03	316.03	328.54	402.18	389.34	325.42	285.77	236.25	240.10	214.25
45	405.00	412.51	425.92	500.00	470.60	410.83	376.65	288.00	295.31	270.00
50	525.00	585.22	566.64	578.81	551.67	490.05	470.60	379.46	389.99	375.70
55	653.45	801.96	723.17	793.90	702.30	618.25	567.84	530.00	552.96	571.26
60	820.82	1008.00	1015.20	1064.96	835.44	828.48	708.74	683.65	756.25	806.73
65	998.51	1371.17	1430.80	1391.60	1031.94	1098.31	1032.26	885.60	1089.54	1184.11
70	1333.08	–	–	–	‐	–	1462.10	1313.83	–	–

*Standard*: If the volume exceeds 1500 m^3^, the mice are considered dead. “–” means the mouse are considered dead.

**TABLE 2:** Xenograft tumor mass.

Mice group	Number	Tumor weight (g)	body weight (g)	10% of body weight (g)
H1299‐shNC	1#	1.3	22	2.2
	2#	0.99	22.5	2.25
	3#	0.91	21.8	2.18
	4#	1.3	20.2	2.02
	5#	1.19	21.5	2.15
	6#	1.14	22.8	2.28
	7#	1.12	20.7	2.07
	8#	1.2	24	2.4
H1299‐shPAICS#1	9#	0.5	22.6	2.26
	10#	0.49	23.8	2.38
	11#	0.5	22.5	2.25
	12#	0.47	21.8	2.18
	13#	0.5	24.1	2.41
	14#	0.47	22.8	2.28
	15#	0.53	20.4	2.04
	16#	0.53	21.6	2.16
H1299‐shPAICS#3	17#	0.42	19.8	1.98
	18#	0.4	21.8	2.18
	19#	0.4	20.2	2.02
	20#	0.42	21.1	2.11
	21#	0.38	21.7	2.17
	22#	0.41	22.7	2.27
	23#	0.4	23.1	2.31
	24#	0.38	23.4	2.34

Seven weeks after transplantation, eight mice were sacrificed in each group. Then, tumors were removed and weighed.

### 2.8 Analysis of coexpressed genes correlate with *PAICS* in NSCLC

**FIGURE 7:**
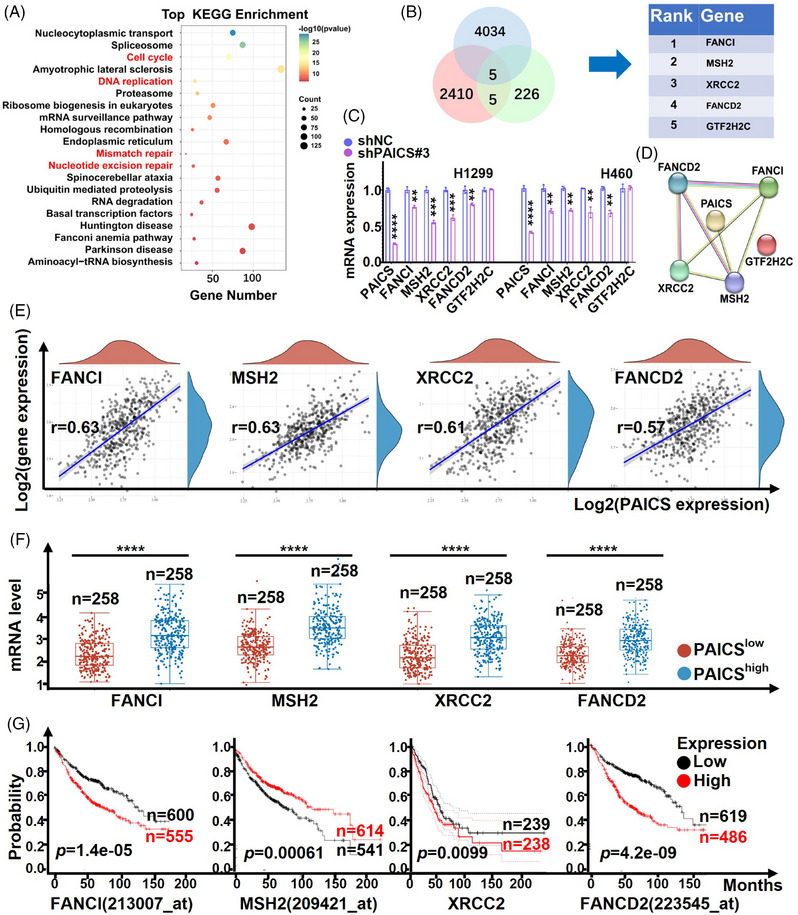
Analysis of coexpressed genes correlated with *PAICS* in NSCLC. (A) KEGG pathway analysis of 4039 *PAICS* coexpressed genes from TCGA database (*n *= 516, *r *= 0.3, *p *< 0.05). (B) Venn diagram depicts the intersection of screening in the above 4039 genes, 2420 DEGs from our own RNA‐seq analysis and 236 DNA damage genes from the TCGA database, five genes were identified and ranked by *p* value. (C) mRNA expression of *FANCI*, *MSH2*, *XRCC2*, *FANCD2*, and *GTF2H2C* in *PAICS*‐knockdown H1299 and H460 cells was detected by qRT‐PCR. (D) The important modules of *PAICS* in the network were analyzed by String. (E) *PAICS* mRNA expression was significantly correlated with *FANCI* (*p *= 2.31e−59, *r *= 0.63), *MSH2* (*p *= 3.23e−58, *r *= 0.63), *XRCC2* (*p *= 1.56e−53, *r *= 0.61), *FANCD2* (*p *= 1.82e−46, *r *= 0.57) confirmed by TCGA database (*n *= 516). (F) The box plot showed the mRNA level of *PAICS* coexpressed genes between PAICS^high^ and PAICS^low^ NSCLC tissues from the TCGA database (*n *= 516). (G) The prognosis value of *PAICS* coexpressed genes for NSCLC patients (data from Kaplan–Meier and TCGA samples). Data are represented as means ± SD. ****p < 0.0001, ***p <  0.001, **p <  0.01, *p < 0.05.

To investigate the *PAICS* coexpressed genes, we identified 4039 potential candidate genes from TCGA database. The 4039 genes were also enriched in cell cycle, DNA replication, mismatch repair, and nucleotide excision repair pathways confirmed by KEGG analysis (Figure 7A). After intersecting the above 4039 genes with 2420 differentially expressed genes related to *PAICS* deletion and 236 DNA damage genes from the TCGA database, five genes were acquired and ranked by *p* value (Figure 7B). The mRNA level of *FANCI*, *MSH2*, *XRCC2*, and *FANCD2* indeed decreased in H1299/H460‐shPAICS cells confirmed by qRT‐PCR (Figure 7C). Complementarily, a notable augmentation in the mRNA expression of FANCI, MSH2, XRCC2, and FANCD2 was observed in H1299/H460‐PAICS‐OE cellular models (Figure S5E). At the same time, *FANCI*, *MSH2*, *XRCC2*, *FANCD2*, and *GTF2H2C* were used to construct a protein‐protein interaction network using String (Figure 7D), which confirming *FANCI*, *MSH2*, *XRCC2*, and *FANCD2* were correlated with PAICS again. Further bioinformatics analysis revealed *FANCI*, *MSH2*, *XRCC2*, and *FANCD2* were positively coexpressed with *PAICS* (Figures 7E and F), and the upregulated expression of them was significantly associated with a poorer prognosis in NSCLC (Figure 7G). All in all, *FANCI*, *MSH2*, *XRCC2*, and *FANCD2* were worthy of further study.

## 3 DISCUSSION

The purpose of our article is to identify therapeutic target(s) for NSCLC patients lacking *EGFR*‐sensitizing mutations.^12^ This subgroup of patients currently lacks effective targeted therapy for carrying neither *EGFR* mutation nor other receptor tyrosine kinase mutations, somehow like triple‐negative breast cancers.^21^ Hence, in this study, a genome‐wide CRISPR/Cas9 knockout screen was performed in *EGFR* wild‐type NSCLC and normal lung cell lines to identify therapeutic targets for this subtype of NSCLC. By means of bioinformatics analysis, three candidate targets, including *PAICS*, were identified as important regulators involved in the progression of *EGFR* wild‐type NSCLC.

**FIGURE 8:**
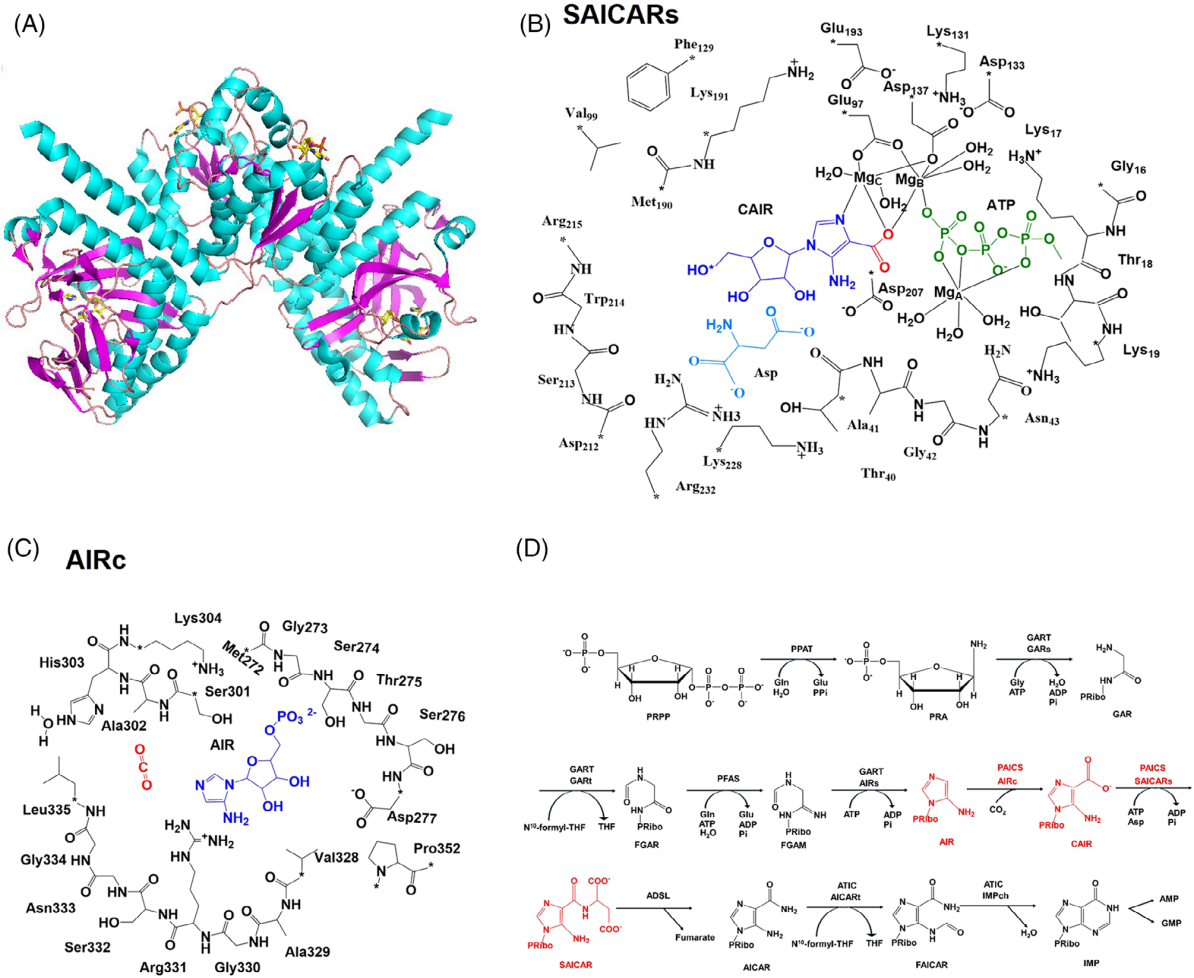
The structure and function of *PAICS*. (A) Molecular structure of human *PAICS*. (B and C) Schematic illustration of the active site models. Carbon atoms labeled with “*” were kept fixed during the geometry optimizations. (D) Scheme of the de novo purine biosynthesis pathway in humans. The reactions catalyzed by *PAICS* are highlighted in red. The following abbreviations are used: PRPP, phosphoribosylpyrophosphate; PRA, phosphoribsylamine; GAR, glycinamide ribonucleotide; FGAR, N‐formylglycinamide ribonucleotide; FGAM, N‐formylglycinamidine ribonucleotide; AIR, aminoimidazole ribonucleotide; CAIR, carboxyaminoimidazole ribonucleotide; SAICAR, N‐succinocarboxamide‐5‐aminoimidazole ribonucleotide; AICAR, aminoimidazole‐4‐carboxamide ribonucleotide; FAICAR, 5‐formamido‐4‐imidazolecarboxamide ribonucleotide; IMP, inosine monophosphate.

*PAICS* is a bifunctional metabolic enzyme catalyzing the conversion of AIR into SAICAR in the de novo purine biosynthesis (Figure 8).^19^ Biosynthesis of purine nucleotides, performed through de novo and salvage pathways, have indispensable and diverse functions inside cells.^22^ De novo pathway employs simple precursors like glycine, glutamine, and aspartate to generate purine nucleotides, which is the main route in highly purine‐producing tissues.^23^ In contrast, the salvage pathway in which nucleoside‐5′‐monophosphates are synthesized from purine nucleobases is important in tissues with low demand for purines.^24^ Rapidly dividing cancer cells rely heavily on purines derived from the de novo pathway, whereas normal cells favor the more economical and less energy‐consuming salvage pathway.^25^ Some tumors even entirely rely on the de novo purine biosynthesis and lack the salvage pathway for adenine nucleotide synthesis.^26^ Enzymes involved in the de novo purine biosynthesis pathway showed high activity in tumors, like dihydrofolate reductase and thymidylate synthase.^27^, ^28^ Therefore, interference of enzymes catalyzing purine metabolism in the de novo pathway has been the target of rational anticancer drugs.^27^

The effects of *PAICS* are complicated and contradictory in different tumor types. *PAICS* is overexpressed in glioma, breast cancer, pancreatic cancer, gastric cancer, prostate cancer, bladder cancer, and colorectal cancer, and its high expression is closely related to the poor survival of tumor patients.^29^, ^30^, ^31^, ^32^, ^33^, ^34^ However, a recent paper by Kobayashi et al.^29^ analyzed 1659 colorectal tumor samples demonstrated a reduction of *PAICS* due to genetic deletion of chromosome 4q during colorectal tumor progression and metastasis. Correspondingly, loss of *PAICS* is also correlated with poor relapse‐free survival in stage III colorectal tumor patients.^29^ Thus, here we set out to perform a pan‐cancer analysis of *PAICS*. Our comprehensive analysis demonstrated that mutations in PAICS have been identified in more than 30 different types of tumors. What is more, cancer patients with genetic alteration of PAICS showed a poor prognosis in OS, PFS, DSS, and DFS. Our results indicated that PAICS expression is elevated in most tumors, with the notable exception of KIRP, where it is lower compared with respective control tissues. Taken together, these results demonstrated the critical role of *PAICS* in tumor progression, suggesting that PAICS is a potential biomarker for predicting the prognosis of tumor patients. However, there are no specific *PAICS*‐targeting agents thus far. Gratifyingly, crystal structure of PAICS with CAIR/SAICAR bound in the active sites was recently revealed, disclosing important structural information for the rational design of *PAICS* inhibitors as potential anticancer drugs.^35^ For lung cancer, amplification of *PAICS* gene and/or overexpression of its protein is significantly associated with poor prognosis of lung adenocarcinoma.^36^, ^37^, ^38^ However, there still have no published papers to report the role and mechanism of *PAICS* on *EGFR* wild‐type NSCLC, which is in a position where no targeted drugs are available. In this study, bioinformatics analysis identified that *PAICS* was upregulated in *EGFR* wild‐type NSCLC cells. According to the patient‐derived tissue microarray analysis in this study, PAICS expression was upregulated in the NSCLC tissue samples and particularly associated with NSCLC progression. Moreover, elevated level of *PAICS* was markedly related to the poor OS of NSCLC patients. All the above results suggested that *PAICS* plays a vital role in *EGFR* wild‐type NSCLC progression, implying *PAICS* might a therapeutic target for it.

As a key regulatory enzyme involved in the de novo purine biosynthesis pathway, *PAICS* serves an important role in regulating cell proliferation, growth, and apoptosis. *PAICS* promotes tumor cells proliferation and migration, whereas the knockdown of *PAICS* leads to tumor cell cycle arrest and apoptosis.^30^, ^39^ In this study, shRNA‐mediated *PAICS* knockdown showed significant suppression on the viability and proliferation, while induction on apoptosis of *EGFR* wild‐type NSCLC cells. Moreover, *PAICS* knockdown resulted in the inhibition of tumor growth and improved OS in a murine xenograft model. Additionally, the augmented expression of PAICS facilitated the proliferation and growth of BEAS‐2B cells and *EGFR* wild‐type NSCLC cells. However, no noticeable changes were observed in *EGFR* mutation NSCLC cells following *PAICS* knockdown.

So, why is the suppression of PAICS expression predominantly efficacious in *EGFR* wild‐type NSCLC? The *EGFR* signaling cascade is instrumental in orchestrating cellular growth and proliferation dynamics, and its inhibition markedly attenuates the proliferation potential of a diverse spectrum of neoplastic cells.^40^, ^41^ We speculated that the activation of subsequent prosurvival pathways, attributed to *EGFR* mutations that amplify the kinase functionality of *EGFR*, induces a compensatory response negating the effects of *PAICS* suppression. For instance, activation of *EGFR* downstream PI3K/AKT pathway contributes to both de novo and salvage purine nucleotide synthesis.^42^ *EGFR* pathway plays a contributory role in both de novo and salvage pathways of purine nucleotide synthesis.^40^, ^41^, ^43^ Inhibition of *PI3K/AKT* reduced de novo synthesis by 75% in logarithmically growing C2C12 mouse mesenchymal cells.^42^ Analogously, the inhibition of *AKT* mediated by MK2296 elicited a 73% decrement in purine biosynthesis within HeLa cellular models.^44^ To reveal the mechanism that the EGFR wild‐type NSCLC cells were more sensitive to the PAICS intervention, we performed the following experiments: first, we identified that the level of IMPDH2, a key enzyme in the de novo purine biosynthesis pathway, is significantly lower in EGFR mutant NSCLC cells than EGFR wild‐type NSCLC cells. This finding suggests that EGFR wild‐type NSCLC cells were more dependent on the de novo purine biosynthesis pathway than the mutant cells. Consequently, the inhibition impact of silencing PAICS was more severe on the EGFR wild‐type than on the mutant NSCLC cells. Second, the PI3K/AKT signaling remained highly activated even after the PAICS gene was knockdown in EGFR mutant NSCLC cells, which may lead to the elevation of the purine salvage synthesis pathway. Furthermore, treating EGFR mutant NSCLC cells with a pan‐PI3K inhibitor BKM120 leads to a significant drop of the proliferation ability of PAICS knockdown cells, suggesting the activated PI3K/AKT signaling could compromise the effect of PAICS knockdown. Therefore, we speculate that targeting PAICS is primarily effective in WT‐EGFR NSCLC due to two factors: first, EGFR wild‐type NSCLC cells were heavily dependent on the de novo purine synthesis pathway; second, the activation of AKT in EGFR mutant NSCLC cells compensates the blockage of de novo purine synthesis pathway by PAICS silencing. Moreover, it was further confirmed that the three major characteristics of malignant tumors (EMT, coagulation, and angiogenesis) dramatically decreased in *PAICS* deficiency *EGFR* wild‐type NSCLC cells. Collectively, *PAICS* could be a highly promising target in *EGFR* wild‐type NSCLC.

We further explored the detailed mechanism underlying *PAICS*‐mediated promoting roles in tumor cell proliferation and survival. The cell cycle pathway plays a significant role in cell growth and survival.^46^ Aberrancy of the cell cycle is one of the most frequent alterations during tumor progression.^47^ Therefore, blocking cell cycle progression is regarded as an effective strategy for eliminating cancer cells.^48^ Some metabolic enzymes periodically translocate to the nucleus to oversee cell cycle regulators' expression.^49^ *GAPDH* promotes cell cycle progression by inducing advancement of the Cyclin *B‐CDK1* peak and delaying the degradation of telomeres.^50^ Pyruvate kinase M2 (*PKM2*) translocation accelerates cell cycle progression by inducing c‐Myc expression.^51^ In the present study, *PAICS* was tightly linked to cell cycle both in single‐cell analysis from CancerSEA and RNA sequencing analysis based on *PAICS* knockdown‐caused differentially expressed genes. Four cell cycle‐related gene sets (G2/M checkpoint, E2F targets, mTOR signaling and mitotic) were included among the nine significantly expressed hallmark gene sets related to *PAICS*. Furthermore, *PAICS* deficiency caused a strong arrest at the G1 phase of *EGFR* wild‐type NSCLC cells, accompanied by the decreased expression of cell cycle factors, including CDK2, CDK6, Cyclin D1, Cyclin E, and p‐Rb expression. Cyclin D‐CDK4/6 complex promotes cell cycle progression in early G1 to late G1. In early G1 phase, Cyclin D‐CDK4/6 phosphorylated Rb at S807/811 to release the Rb from E2F, which activates expression of genes that regulate entry into S phase.^52^ Cyclin E is one of E2F target genes. As Cyclin E‐CDK2 phosphorylates Rb, Cyclin E reinforces its own expression through a positive feedback loop.^53^ Moreover, Cyclin E‐CDK2 carried out a series of sequential phosphorylations to trigger DNA replication at the onset of the S phase.^54^ Therefore, downregulation of CDK2, CDK6, Cyclin D1, Cyclin E, and p‐Rb will cause cell cycle arrest at the G1 phase, which are consistent with our results. Evidently, *PAICS* knockdown‐induced cell cycle arrest might play a critical role in the survival of *EGFR* wild‐type NSCLC cells. Since PKM2 is activated by SAICAR, the metabolite of *PAICS*, it is reasonable to assume that *PAICS* is involved in cell‐cycle control by regulating the translocation of PKM2.

Precise DNA replication during the S phase is necessary for cell‐cycle progression.^55^ If DNA damage is present, DNA‐repair mechanisms are triggered to arrest the cell cycle until the damaged DNA is repaired.^56^ While severe DNA damage beyond repair will lead to cell death or senescence.^57^ Thus, DNA damage‐induced apoptosis is a viable therapeutic target for tumors. Huang et al. have reported that PAICS participates in DNA damage repairing by targeting HDAC1‐DAD51 in gastric cancer.^19^ In this study, *PAICS* deficiency induced DNA damage in *EGFR* wild‐type NSCLC cells, which is consistent with the role of *PAICS* in gastric cancer. We further screened out five *PAICS* coexpression genes as *FANCI*, *MSH2*, *XRCC2*, *FANCD2*, and *GTF2H2C* using an integrated bioinformatics analysis. Further studies confirmed that there was a significant positive correlation in expression of *FANCI*, *MSH2*, *XRCC2*, *FANCD2*, and *PAICS*. Moreover, high level of *FANCI*, *MSH2*, *XRCC2*, *and FANCD2* was also significantly associated with poorer prognosis in NSCLC. Therefore, we speculated that PAICS might regulate DNA damage by interacting with the *FANCI*, *MSH2*, *XRCC2*, and *FANCD2*. The detailed mechanism of *PAICS* in regulating *FANCI*, *MSH2*, *XRCC2*, and *FANCD2* remains unclear and needs further exploration.

In conclusion, we successfully identified *PAICS* as a potential therapeutic target for *EGFR* wild‐type NSCLC via a genome‐wide CRISPR/Cas9 screen. As a novel oncogene, *PAICS* deficiency promotes *EGFR* wild‐type NSCLC apoptosis by inducing DNA damage both in vitro and in vivo. These findings confirmed that *PAICS* is an essential gene and provided a novel and feasible therapeutic solution for NSCLC patients carrying wild‐type *EGFR*, which are not suitable for current targeted therapies.

## 4 MATERIALS AND METHODS

### 4.1 Cell lines and culture

Human NSCLC cell lines (H460, A549, H1299, PC9, H1975) and 293T cells were obtained from the Cell Bank of the Chinese Academy of Sciences (Shanghai, China). Human normal bronchial epithelial cell line BEAS‐2B was a gift from Dr. Y. Sun (Nanjing Medical University, Jiangsu, China). A549 and BEAS‐2B cell lines were maintained in DMEM (Gibco‐Invitrogen, CA, USA), whereas H460, H1299, PC9 and H1975 cells were cultured in RPMI‐1640 (Gibco‐Invitrogen) supplemented with 10% fetal bovine serum (VACCA, Shanghai, China) and penicillin (100 U/mL)–streptomycin (100 µg/mL) less than 15 passages, and authenticated by Biowing Biotech (Shanghai, China). These cell lines were cultured in a humid incubator (5% CO_2_, 37°C).

### 4.2 Vector construction and lentiviral infection

The *PAICS* knockdown and the scramble control lentiviral plasmids (shPAICS‐1 or shPAICS‐3, shNC) were obtained from Tsingke Biotechnology (Beijing, China). shRNA sequences targeting PAICS (shPAICS#1: CCGGGACCAGATTACAGCAGGAAATCTCGAGATTTCCTGCTGTAATCTGGTCTTTTTT; shPAICS#3: CCGGCGCAGTGTGAAATGATTCCAACTCGAGTTGGAATCATTTCACACTGCGTTTTTT) were cloned into plko.1‐copGFP‐2A‐PURO vectors to achieve PAICS knockdown vectors. 293T cells were seeded into several six‐well plates (5 × 10^5^ cells/well) for 24 h before transfection. When 293T cell density reaches 60–70%, a mixture containing 1 mL of shPAICS/shNC Plasmid and 3 µL of polybrene (1:1000) was transfected to each well of 293T cells. The cells were replenished with a fresh medium after 18 h posttransfection. After another 48 h, puro (0.5 µg/mL for BEAS‐2B cells, 1 µg/mL for H1299 and A549 cells, 2 µg/mL for H460 cells, PC9 cells and H1975 cells) was added to the cells until a complete eradication of cells in the control group was observed. 48 h postwithdrawal of puromycin, total RNA and protein were extracted from the meticulously selected cells and subjected to qRT‐PCR and Western blot detection.

Overexpressed *PAICS* lentiviral vectors (*PAICS‐*OE) and control group (NC) were constructed based on human *PAICS* sequences from the Ensembl Release 110 (July 2023) (gene nos. ENST00000505164) and synthesized by the GeneCopoeia, Inc. *PAICS* overexpressing sequences were cloned into pEZ‐Lv242 vectors to produce *PAICS*—overexpressing vectors. 293T cells were seeded into several six‐well plates (5 × 10^5^ cells/well) for 24 h before transfection. When 293T cell density reaches 60−70%, a mixture containing 1 mL of PAICS‐OE/NC Plasmid and 3 µL of polybrene (1:1000) was transfected to each well of 293T cells. The cells were replenished with a fresh medium after 18 h posttransfection. After another 48 h, puro (0.5 ug/mL for BEAS‐2B cells, 1 ug/mL for H1299 and A549 cells, 2 ug/mL for H460 cells) was added to the cells until a complete eradication of cells in the control group was observed. 48 h postwithdrawal of puromycin, total RNA and protein were extracted from the meticulously selected cells and subjected to qRT‐PCR and Western blot detection, respectively.

### 4.3 CRISPR/Cas9 screening with LentiCRISPRv2 library

Genome‐wide CRISPR knockout screening was performed as described.^58^, ^59^ As the GeCKO v2.0 library contains 123411 sgRNAs targeting 19,050 genes, about 1.6 × 10^8^ NCI‐H460, A549, and BEAS‐2B cells need to be infected to achieve 300‐fold coverage at a multiplicity of infection of 0.3–0.5. Cells were seeded in 12‐well plates for 24 h and then infected with lentivirus by centrifuging at 450 *g* for 2 h at 37°C in the presence of 8 µg/mL polybrene to increase transduction efficiency. After the spin, cells were replenished with fresh media (without polybrene) and cultured for another 24 h. Then, those cells were cultured in 15‐cm dishes to an appropriate density. Puromycin was then added into cells for 7 days at 1 µg/mL for A549 and BEAS‐2B and 2 µg/mL for NCI‐H460 cells. 4 × 10^7^ cells were harvested and stored at −80°C for subsequent genomic DNA (gDNA) isolation (as Day 0). Meanwhile, other 4 × 10^7^ cells were cultured every 3 days to maintain adequate sgRNA library complexity. On the 14th day, 4 × 10^7^ cells were collected for gDNA isolation and next‐generation sequencing (as Day 14). MAGeCKFlute was used to analyze and identify candidate genes.

### 4.4 Cell proliferation assay

Cell proliferation was assessed using the Cell Counting Kit‐8 (CCK8; Yeasen, Shanghai, China). A549, H460, H1299, BEAS‐2B, PC9 and H1975 cells (1 × 10^3^ cells/well) were seeded into a 96‐well plate and severally cultured for 24, 48 and 72 h. The absorbance was measured at 450 nm using a multi‐function microplate reader (Fluoroskan Ascent FL analyzer) after cells were incubated with CCK8 at 37°C for 3 h.

### 4.5 Colony formation assay

A549, H460, H1299, BEAS‐2B, PC9, and H1975 cells (500 cells/well) were seeded into 12‐well plates and incubated for 14 days at 37°C with 5% CO_2_ in a humidified incubator. After incubation, the culture medium was discarded, and cells were washed with PBS. Finally, the cells were fixed with 4% paraformaldehyde and stained with a Giemsa Stain solution. Finally, all colonies were manually scored.

### 4.6 Cell cycle analysis and apoptosis assay

For analysis of the cell cycle, H1299, A549, and H460 cells were seeded in six‐well plates and incubated with different treatments. Cells were washed twice in ice‐cold PBS and fixed in 75% cold ethanol overnight at 4°C. After removing the ethanol, the fixed cells were washed with pre‐cold PBS twice, treated with 0.1 mg/mL RNase A at 37°C in the dark for 30 min, and stained with 50 µg/mL of propidium iodide (PI) (Sigma–Aldrich, St Louis, USA) solution in the dark for 5 min. Finally, the stained cells were analyzed with a FACS Calibur flow cytometer.

For apoptosis assay, H1299, A549, and H460 cells in the logarithmic growth phase were seeded in 6‐well plates, then incubated at 37°C and 5% CO_2_ condition. After incubation, the cells were rinsed with pre‐cold PBS twice and then stained with Annexin V and PI according to the manufacturer's instructions. A flow cytometer was used to analyze the stained cells within 1 h.

### 4.7 Western blot

Proteins were extracted from A549, H460, H1299, BEAS‐2B, PC9, and H1975 cells by RIPA lysis buffer (Beyotime Biotechnology, Shanghai, China) containing 0.1 M PMSF and protease inhibitors (Roche, Basel, Switzerland). Proteins were quantified by the BCA Protein Assay Kit (Yeasen). Proteins were fractionated by SDS‐PAGE, and then transferred to PVDF membranes (Merck Millipore, MA, USA), and blocked with 3% bovine serum albumin for 2 h at room temperature (RT). The membranes were probed with primary antibodies at 4°C overnight. The following primary antibodies were used for immunoblotting: PAICS (ProteinTech; no. 12967‐1‐AP), GAPDH (ProteinTech; no. 60004‐1‐lg), Cyclin E (Santa‐Cruze; no. sc‐377100), Cyclin D1/CDK2/CDK6 (Cell Signaling Technology; Cell Cycle Regulation Antibody Sampler Kit, no. 9932T), Phospho‐Rb (Cell Signaling Technology; no. 8516S). Then, the membranes were washed by TBST (1×) and incubated with a corresponding horseradish peroxidase‐conjugated secondary antibody for 2 h at RT. The immunoreactive bands were visualized using an ECL kit (Beyotime) and analyzed using Imager software (Tanon, Shanghai, China).

### 4.8 Immunofluorescence assay

A549, H460, H1299, and BEAS‐2B cells cultured on confocal dishes were fixed with 4% paraformaldehyde (Sigma) for 15 min at RT and permeabilized with 0.1% Triton X‐100 for 15 min at 37°C. After blocking with 10% goat serum for 1 h at RT, cells were incubated with the primary antibodies against PAICS Rabbit IgG (ProteinTech; 1:100 dilution) at 4°C overnight. The next day, cells were incubated with corresponding secondary antibodies against Cy3 Anti‐Rabbit IgG (Absin; 1:100 dilution) combined with FITC‐γH2AX Mouse IgG (Biolegend, 1:100 dilution) at RT for 90 min. 4′,6‐diamidino‐2‐phenylindole (Yeasen Biotechnology) was then applied to stain the nuclei. Finally, the images of immunofluorescence were obtained with the confocal laser‐scanning microscope (Carl Zeiss, Jena, Germany).

### 4.9 RNA isolation and qRT‐PCR

Total RNA was extracted from H460 and H1299 cells by Trizol reagent (Invitrogen) and used to synthesize cDNA with the PrimeScriptTM RT reagent Kit (TaKaRa, Dalian, China) according to the manufacturer's instructions. qRT‐PCR was performed on an ABI 7900HT PCR sequencer (Applied Biosystems, Massachusetts, USA) using TB Green® Premix Ex Taq™ II (TaKaRa). Primers sequences used for qRT‐PCR were as follows: *FANCI*, forward: 5′‐CCACCTTTGGTCTATCAGCTTC‐3′, reverse: 5′‐CAACATCCAATAGCTCGTCACC‐3′. *GAPDH*, forward: 5′‐TGCACCACCAACTGCTTAGC‐3′, reverse: 5′‐GGCATGGACTGTGGTCATGAG‐3′. *MSH2*, forward: 5′‐CACTGTCTGCGGTAATCAAGT‐3′, reverse: 5′‐CTCTGACTGCTGCAATATCCAAT‐3′. *XRCC2*, forward: 5′‐TGCTTTATCACCTAACAGCACG‐3′, reverse: 5′‐TGCTCAAGAATTGTAACTAGCCG‐3′. *FANCD2*, forward: 5′‐AAAACGGGAGAGAGTCAGAATCA‐3′, reverse:5′‐ACGCTCACAAGACAAAAGGCA‐3′. *GTF2H2C*: forward: 5′‐TTCCGGCTGAGAGTCCTTCT‐3′, reverse: 5′‐TCCTCCTTCCCTATGAGCCC‐3′. Fold changes in mRNA expression were calculated using 2^−ΔΔCt^ ^60^ and standardized based on *GAPDH*.

### 4.10 Tumor xenograft model

Mice were housed and handled according to institutional guidelines complying with local legislation. All experiments with animals were approved by the Nanjing Medical University Animal Welfare and Ethics Committee (Ethical approval number: SYXK(Su)‐2020‐0022). All BALB/C mice were purchased from Jiangsu GemPharmtech co., Ltd (Nanjing, China) and were adapted to the environment for a week before the experiment. To determine the role of *PAICS* in vivo, H1299 cells (5 × 10^6^ cells/mouse) with stable knockdown of PAICS and the corresponding control were injected subcutaneously into the mice (18 mice/group). Tumor size was measured every 5 days and calculated using the following formula: volume  =  (length × width^2^) × 0.5. After 7 weeks, 8 mice/group were sacrificed, and tumors were removed, weighed, and fixed for immunohistochemistry detection of Ki67 (Servicebio; GB111499). Once the tumor volume reached 1800 mm^3^, mice were sacrificed by cervical dislocation and recorded as dead.

### 4.11 Tissue microarray and IHC

Lung cancer (HLugA180Su02) was purchased from Outdo Biotech (Shanghai, China). Patients who lacked paired nontumor tissues or had tissue flaking, or failed to follow up were excluded. IHC staining was performed as previously described. PAICS antibody (ProteinTech; no. 12967‐1‐AP) was used as the primary antibody.

### 4.12 Gene expression analysis using publicly available datasets

Gene expression levels of *PAICS* in tumors and adjacent normal samples were obtained from UALCAN (http://ualcan.path.uab.edu), Gepia (http://gepia2.cancer‐pku.cn/#analysis), and TNMplot (https://tnmplot.com/analysis/). The Kaplan–Meier analysis was performed with the data downloaded from Kaplan–Meier Plotter (http://kmplot.com/analysis/). CancerSEA (http://biocc.hrbmu.edu.cn/CancerSEA/) which depicts single‐cell functional status maps was used to analyze the roles of *PAICS*. Protein–protein interaction networks were analyzed by String (https://cn.string‐db.org/). The “Top KEGG Enrichment” terms were analyzed with the R analysis (R version 4.2.2) using the TCGA database.

### 4.13 RNA sequencing analysis and gene annotation

Whole RNA was extracted the same as qRT‐PCR. RNA samples were subjected to RNA sequencing (RNA‐seq) analysis on the BGISEQ‐500 system by Beijing Genomics Institute, China.

### 4.14 *PAICS* genetic alteration and gene expression analysis

Data regarding alteration frequency, mutation type, mutated site information, and copy number alterations of PAICS across all TCGA tumors were obtained using the cBioPortal tool (https://www.cbioportal.org/). We compared OS and DFS data for TCGA cancer types, categorizing them based on the presence or absence of PAICS genetic alterations. The Tumor Immune Estimation Resource platform (TIMER2, version 2, http://timer.cistrome.org/) facilitated our analysis of *PAICS* expression profiles in various tumor types and their adjacent normal tissues as sourced from TCGA.

### 4.15 Enzyme‐linked immunosorbent assay

The level of IMPDH2 in H1299, A549, PC9, and H1975 cell supernatants were assessed by ELISA kit (no. ELK4812; ELK Biotechnology, Wuhan, China) in line with the protocol of manufacturer.

### 4.16 Statistical analysis

Statistical analysis was performed using GraphPad Prism software (9.0; GraphPad Software, Inc.). Each experiment was repeated three times. Data are presented as the mean ± standard deviation. Unpaired Student's *t*‐test was used for two‐group comparisons, and one‐way ANOVA followed by Tukey's post hoc test was used for multiple comparisons. *p* < 0.05 was considered to indicate a statistically significant difference.

## AUTHOR CONTRIBUTIONS

Yufeng Li and Lingyun Zhu performed most of the experiments. Jiaqi Mao, Hongrui Zheng, Ziyi Hu, Suisui Yang, Tianyu Mao, Tingting Zhou, Pingping Cao, Hongshuai Wu, and Xuerong Wang searched the literature and conceived and supervised all the studies. Jing Wang, Fan Lin, and Hua Shen conceived and designed the research, participated in the writing and manuscript revision. All authors have read and approved the final manuscript.

## CONFLICT OF INTEREST STATEMENT

The authors declare that they have no competing interests.

## ETHICS STATEMENT

This research was conducted under the stringent oversight and approval of the Nanjing Medical University Animal Welfare and Ethics Committee (Ethical approval number: SYXK(Su)‐2020‐0022).

## PATIENT CONSENT FOR PUBLICATION

Not applicable.

## Supporting information

Supporting information

Supporting information

## Data Availability

The datasets used and/or analyzed during the present study are available from the corresponding author on reasonable request.
