# Supplementary material for: Genome‐scale CRISPR–Cas9 screen identifies PAICS as a therapeutic target for EGFR wild‐type non‐small cell lung cancer
Source: MedComm (2020). 2024 Mar 9;5(3):e483. doi: 10.1002/mco2.483 (PMC10924642; doi:10.1002/mco2.483)
Supplement: Supplementary file 1 — Supporting information [file MCO2-5-e483-s001.docx]

**Genome-scale CRISPR-Cas9 screen identifies *PAICS* as a therapeutic target for *EGFR* wild-type** **non-small cell lung cancer**

Yufeng Li^1,2^#, Lingyun Zhu^3^#, Jiaqi Mao^3^, Hongrui Zheng^4^, Ziyi Hu^3^, Suisui Yang^3^, Tianyu Mao^1^, Tingting Zhou^3^, Pingping Cao^3^, Hongshuai Wu^3,5^, Xuerong Wang^5^, Jing Wang^3^*, Fan Lin^3,6,7^*, Hua Shen^1,2^*

^1^Department of Medical Oncology, The First Affiliated Hospital of Nanjing Medical University, Nanjing, Jiangsu, China

^2^ Department of Medical Oncology, The Affiliated Sir Run Run Hospital of Nanjing Medical University, Nanjing, Jiangsu, China.

^3^Department of Cell Biology, School of Basic Medical Sciences, Nanjing Medical University, Nanjing, Jiangsu, China

^4^Department of Orthopedics, Taizhou Hospital of Zhejiang Province, Affiliated to Wenzhou Medical University, Zhejiang, China

^5^Department of Pharmacology, Nanjing Medical University, Nanjing, Jiangsu, China

^6^Institute for Brain Tumors & Key Laboratory of Rare Metabolic Diseases, Nanjing Medical University; Nanjing, Jiangsu, China

^7^Department of Gastroenterology, The First Affiliated Hospital, and College of Clinical Medicine of Henan University of Science and Technology, Luoyang, Henan, China

#These authors contributed equally to this work.

*Corresponding Author:

Jing Wang and Fan Lin, Department of Cell Biology, School of Basic Medical Sciences, Nanjing Medical University, 101 Longmian Avenue, Jiangning District, Nanjing, Jiangsu, China.

Hua Shen, Department of Medical Oncology, The First Affiliated Hospital of Nanjing Medical University, Nanjing, Jiangsu, China.

E-mail: [wangjing1124321@njmu.edu.cn](mailto:wangjing1124321@njmu.edu.cn), [linfee@me.com](mailto:linfee@me.com), [medshenhua@sina.com](mailto:medshenhua@sina.com)


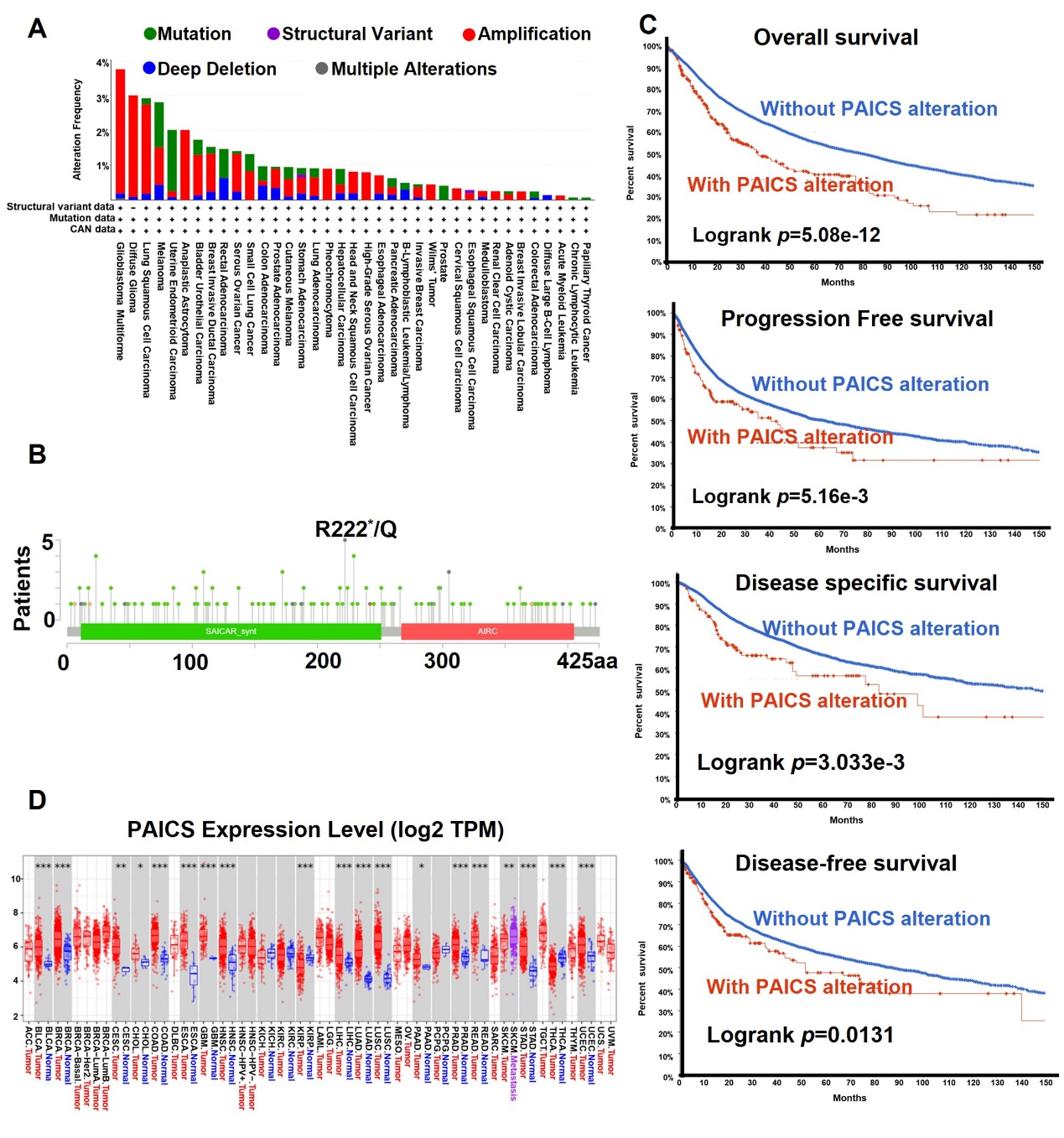


**Figure S1** Mutation status and expression level of PAICS in TCGA tumors. (AB) Mutation status of PAICS in TCGA tumors was analyzed using the cBioPortal tool. The alteration frequency with mutation type (A) and mutation site (B) are displayed. (C) Analysis of the correlation between mutation status and Overall survival, Progression-free survival, Disease-specific survival and Disease-free survival of pan-cancer using the cBioPortal tool. (D) Expression level of PAICS in TCGA tumors vs adjacent tissues (if available) as visualized by TIMER2. **p* < 0.05; ***p* < 0.01; ****p* < 0.001.


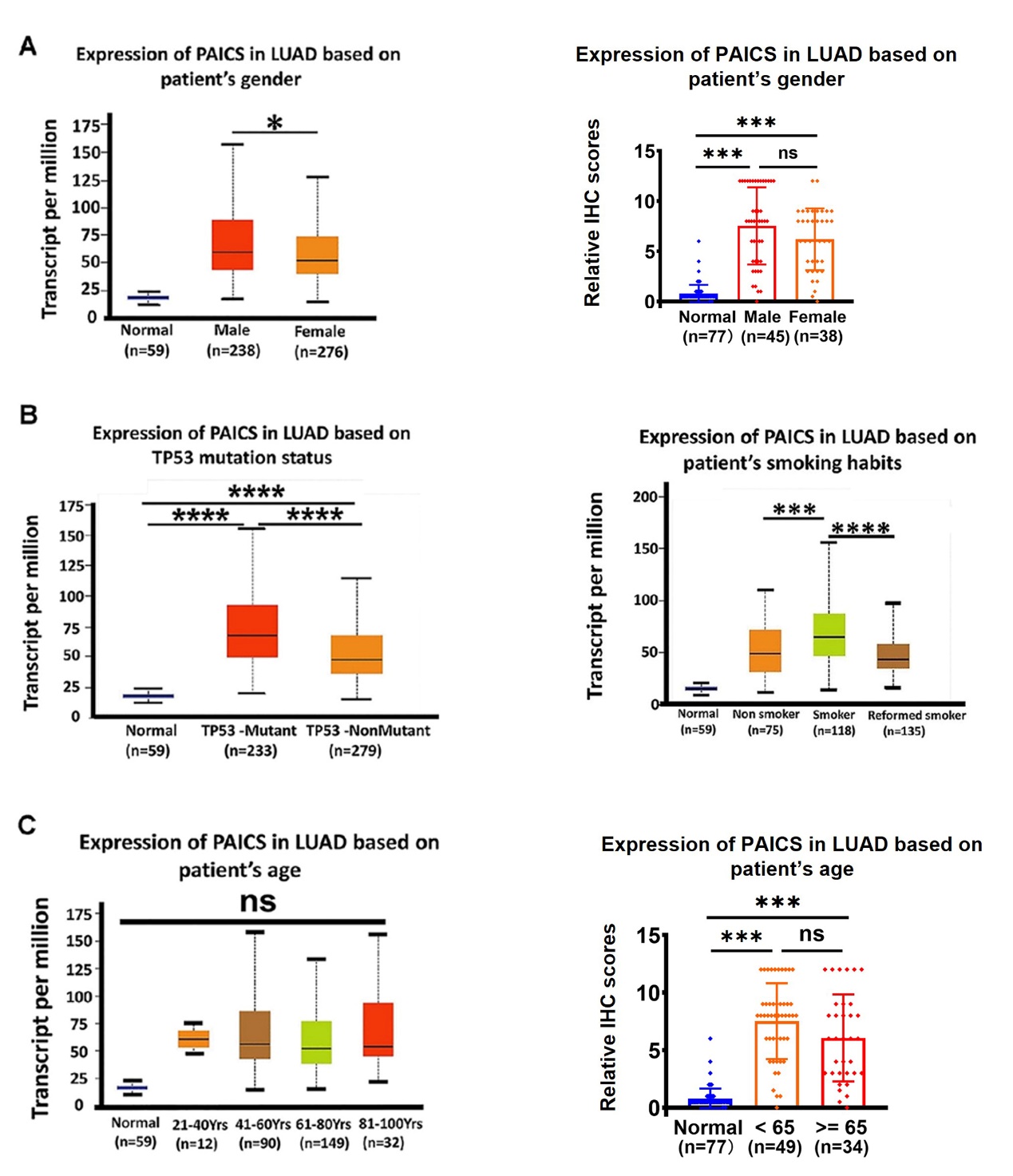


**Figure S2** Relationship between the PAICS level and NSCLC patient's gender (A), TP53 mutation status (B) and age (C) based on the data from TCGA database (left) and patient-derived tissue microarray (right).

**
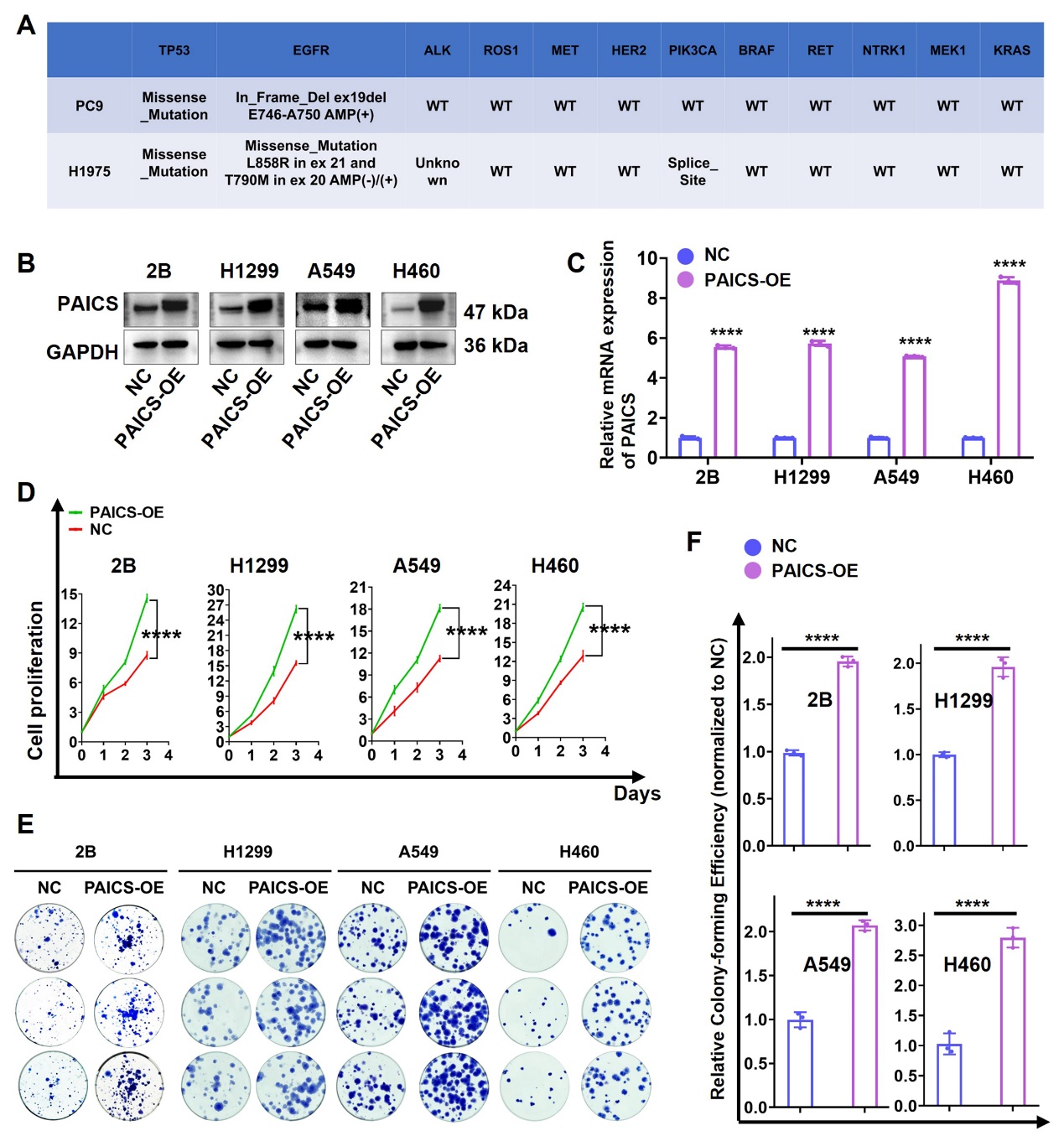
**

**Figure S3** (A) The status of some common driver gene mutations of PC9 and H1975. (BC) Protein and mRNA levels of PAICS in BEAS-2B, H1299, A549, and H460 cells after PAICS-OE lentivirus transfection were detected by Western blot (B) and qRT-PCR (C), respectively. (D) Cell viability of PAICS-OE and NC cells confirmed by CCK8 assays. (EF) Colony formation (E) and Clone quantification (F) of PAICS-OE and NC cells (*n* = 3). Data are represented as means ± SD (*n* = 3). **** *p* < 0.0001.

**
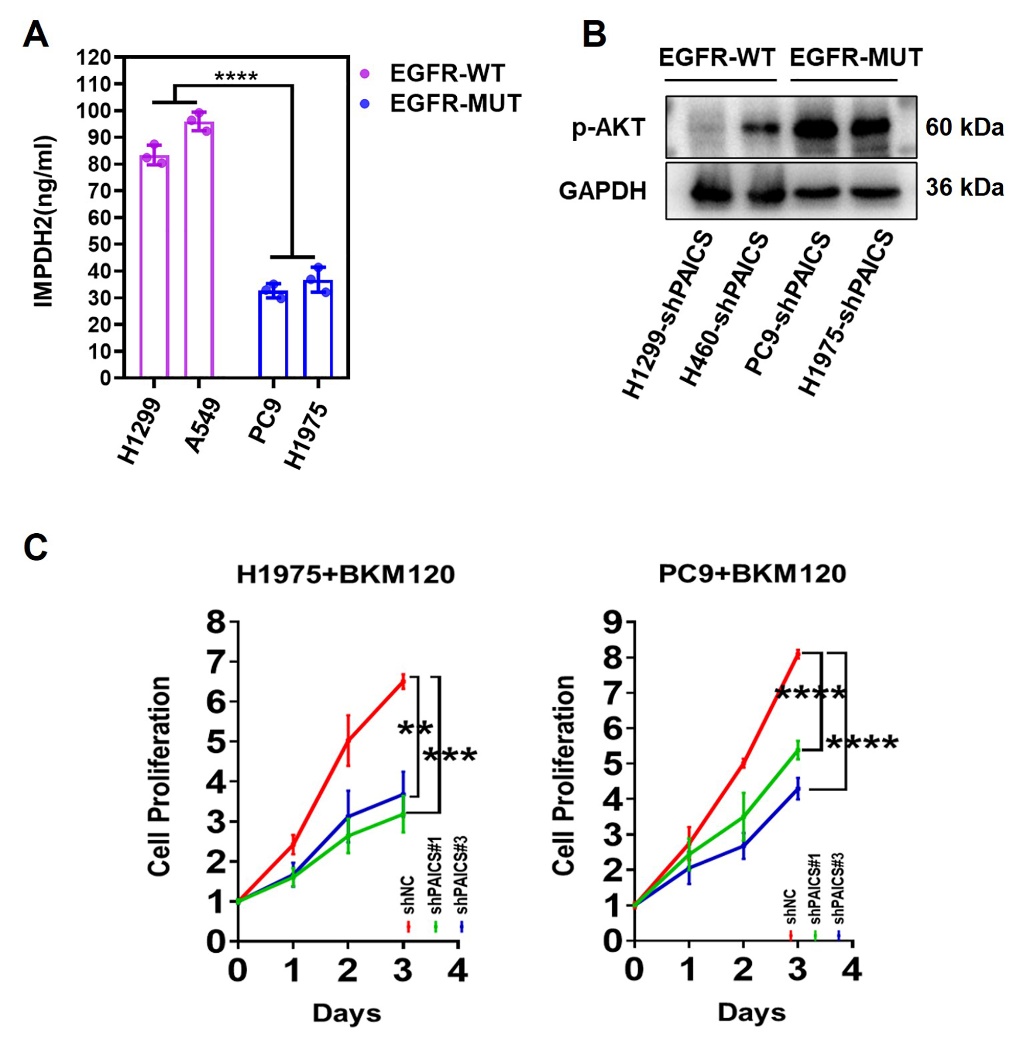
**

**Figure S4** (A) The concentration of IMPDH2 in H1299, A549, PC9 and H1975 cells was determined by ELISA assay. (B) Protein levels of p-AKT in PAICS knockdown H1299, A549, PC9 and H1975 cells were detected by Western blot. (C) Cell viability of BKM120-treated PAICS knockdown H1975 (7 μM BKM120) and PC9 cells (1 μM BKM120) confirmed by CCK8 assays. Data are represented as means ± SD (*n* = 3). *****p* < 0.0001, ***p* < 0.01.

**
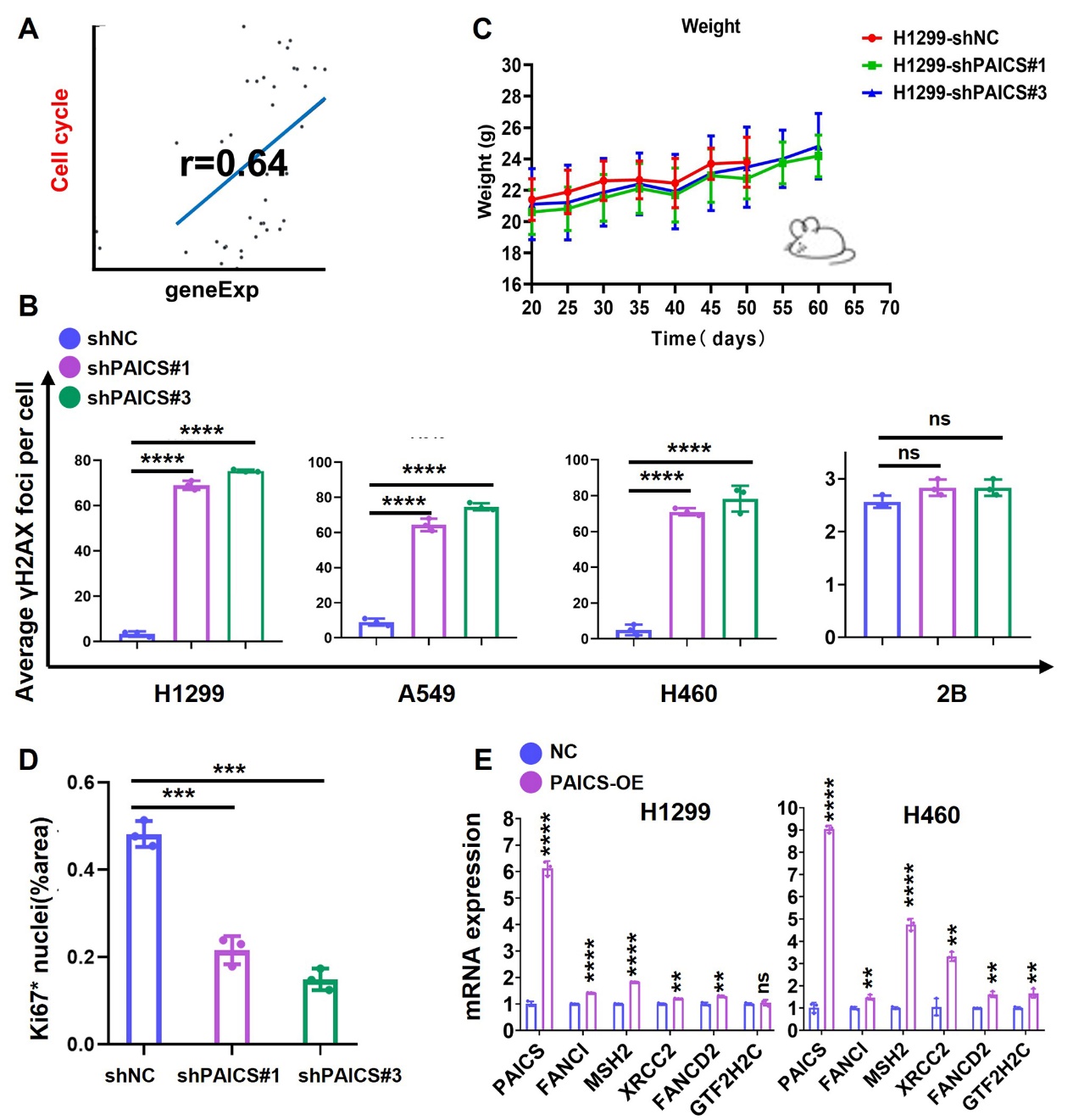
**

**Figure S5 (A)** Relationship between PAICS expression and cell cycle based on single-cell analysis from CancerSEA. (B) Quantification of γH2AX fluorescence intensity in Fig.5F. Data are represented as means ± SD (*n* = 3). **** *p* < 0.0001. (C) Body weight chart of PAICS knockdown and the control mice. Data are represented as means ± SD (*n* = 8). (D) Ki67 Quantitation of the relative Ki67 expression in Fig. 6G. (E) mRNA expression of FANCI, MSH2, XRCC2, FANCD2 and GTF2H2C in PAICS-overexpression H1299 and H460 cells was detected by qPCR. Data are represented as means ± SD (*n* = 3). ***p* < 0.01, ****p* < 0.001, *****p* < 0.0001.

**Table 1. Subcutaneous tumor volume of mice.**

| Time（days） | Tumor volume(mm^3^) | | | | | | | | | |
| --- | --- | --- | --- | --- | --- | --- | --- | --- | --- | --- |
|  | H1299-shNC | | | | | | | | | |
| 0 | 1# | 2# | 3# | 4# | 5# | 6# | 7# | 8# | 9# | 10# |
| 20 | 238.05 | 210.94 | 55.69 | 98.31 | 62.50 | 90.75 | 46.23 | 62.50 | 222.91 | 108.00 |
| 25 | 333.90 | 329.25 | 88.48 | 153.60 | 171.50 | 140.40 | 98.31 | 137.31 | 261.56 | 256.00 |
| 30 | 503.61 | 457.65 | 326.40 | 357.64 | 296.35 | 284.00 | 225.26 | 228.27 | 425.25 | 402.18 |
| 35 | 605.05 | 732.05 | 622.91 | 613.84 | 589.84 | 488.45 | 400.95 | 428.69 | 655.99 | 578.81 |
| 40 | 804.65 | 1031.25 | 739.26 | 906.304 | 744.15 | 622.91 | 525.00 | 530.60 | 972.00 | 800.81 |
| 45 | 1000.80 | 1155.07 | 1106.63 | 1021.21 | 881.37 | 752.64 | 740.60 | 734.22 | 1183.00 | 1098.50 |
| 50 | 1461.25 | 1400.77 | 1333.08 | 1421.00 | 1093.75 | 932.91 | 1132.63 | 1098.50 | 1482.05 | 1431.64 |
| 55 | - | - | - | - | 1280.66 | 1225.25 | 1441.73 | 1482.55 | - | - |

| H1299-shPAICS#1 | | | | | | | | | | |
| --- | --- | --- | --- | --- | --- | --- | --- | --- | --- | --- |
| 0 | 11# | 12# | 13# | 14# | 15# | 16# | 17# | 18# | 19# | 20# |
| 20 | 102.85 | 55.18 | 94.22 | 89.24 | 90.75 | 39.75 | 121.00 | 75 | 50.63 | 32.46 |
| 25 | 129.6 | 88.48 | 134.95 | 117.00 | 124.65 | 62.50 | 188.42 | 132.10 | 111.60 | 74.43 |
| 30 | 205.80 | 175.07 | 193.55 | 191.56 | 183.75 | 111.60 | 274.44 | 163.84 | 166.46 | 130.24 |
| 35 | 311.65 | 273.78 | 266.81 | 272.00 | 262.40 | 194.51 | 355.20 | 215.60 | 225.00 | 191.56 |
| 40 | 433.35 | 406.56 | 382.93 | 402.04 | 340.61 | 256.00 | 496.86 | 275.70 | 295.86 | 300.80 |
| 45 | 575.00 | 550.00 | 543.744 | 520.00 | 465.75 | 368.55 | 614.66 | 370.88 | 388.80 | 387.20 |
| 50 | 704.11 | 739.26 | 729.00 | 611.89 | 595.51 | 485.15 | 681.46 | 478.33 | 546.21 | 531.61 |
| 55 | 876.10 | 907.20 | 970.03 | 740.60 | 715.01 | 612.52 | 912.52 | 635.42 | 665.50 | 704.11 |
| 60 | 1053.26 | 1089.54 | 1264.76 | 878.40 | 864.00 | 807.65 | 1143.07 | 807.65 | 893.10 | 914.40 |
| 65 | 1359.46 | 1461.89 | - | 1106.95 | 1016.06 | 1124.05 | 1440.60 | 1016.06 | 1176.12 | 1139.91 |
| 70 | - | - | - | 1441.73 | - | - | - | - | 1524.31 | - |

| H1299-shPAICS#3 | | | | | | | | | | |
| --- | --- | --- | --- | --- | --- | --- | --- | --- | --- | --- |
| 0 | 21# | 22# | 23# | 24# | 25# | 26# | 27# | 28# | 29# | 30# |
| 20 | 111.93 | 111.01 | 122.79 | 219.38 | 186.62 | 154.87 | 45.86 | 85.18 | 53.14 | 87.64 |
| 25 | 127.83 | 122.40 | 139.54 | 256.00 | 199.84 | 171.50 | 117.00 | 94.77 | 61.37 | 93.75 |
| 30 | 153.76 | 138.92 | 156.78 | 285.89 | 213.75 | 219.49 | 161.84 | 116.06 | 87.12 | 109.55 |
| 35 | 215.60 | 275.68 | 252.05 | 344.61 | 318.03 | 269.00 | 231.23 | 158.44 | 116.25 | 153.00 |
| 40 | 292.03 | 316.03 | 328.54 | 402.18 | 389.34 | 325.42 | 285.77 | 236.25 | 240.10 | 214.25 |
| 45 | 405.00 | 412.51 | 425.92 | 500.00 | 470.60 | 410.83 | 376.65 | 288.00 | 295.31 | 270.00 |
| 50 | 525.00 | 585.22 | 566.64 | 578.81 | 551.67 | 490.05 | 470.60 | 379.46 | 389.99 | 375.70 |
| 55 | 653.45 | 801.96 | 723.17 | 793.90 | 702.30 | 618.25 | 567.84 | 530.00 | 552.96 | 571.26 |
| 60 | 820.82 | 1008.00 | 1015.20 | 1064.96 | 835.44 | 828.48 | 708.74 | 683.65 | 756.25 | 806.73 |
| 65 | 998.51 | 1371.17 | 1430.80 | 1391.60 | 1031.94 | 1098.31 | 1032.26 | 885.60 | 1089.54 | 1184.11 |
| 70 | 1333.08 | - | - | - | - | - | 1462.10 | 1313.83 | - | - |

Standard: If the volume exceeds 1500 m^3^, the mice are considered dead. “-” means the mouse are considered dead.

**Table 2. Xenograft tumor mass.**

| Mice Group | Number | Tumor weight (g) | body weight (g) | 10% of body weight (g) |
| --- | --- | --- | --- | --- |
| H1299-shNC | 1# | 1.3 | 22 | 2.2 |
|  | 2# | 0.99 | 22.5 | 2.25 |
|  | 3# | 0.91 | 21.8 | 2.18 |
|  | 4# | 1.3 | 20.2 | 2.02 |
|  | 5# | 1.19 | 21.5 | 2.15 |
|  | 6# | 1.14 | 22.8 | 2.28 |
|  | 7# | 1.12 | 20.7 | 2.07 |
|  | 8# | 1.2 | 24 | 2.4 |
| H1299-shPAICS#1 | 9# | 0.5 | 22.6 | 2.26 |
|  | 10# | 0.49 | 23.8 | 2.38 |
|  | 11# | 0.5 | 22.5 | 2.25 |
|  | 12# | 0.47 | 21.8 | 2.18 |
|  | 13# | 0.5 | 24.1 | 2.41 |
|  | 14# | 0.47 | 22.8 | 2.28 |
|  | 15# | 0.53 | 20.4 | 2.04 |
|  | 16# | 0.53 | 21.6 | 2.16 |
| H1299-shPAICS#3 | 17# | 0.42 | 19.8 | 1.98 |
|  | 18# | 0.4 | 21.8 | 2.18 |
|  | 19# | 0.4 | 20.2 | 2.02 |
|  | 20# | 0.42 | 21.1 | 2.11 |
|  | 21# | 0.38 | 21.7 | 2.17 |
|  | 22# | 0.41 | 22.7 | 2.27 |
|  | 23# | 0.4 | 23.1 | 2.31 |
|  | 24# | 0.38 | 23.4 | 2.34 |

Seven weeks after transplantation, 8 mice were sacrificed in each group. Then tumors were removed and weighed.
